# Supplementary material for: DNA Barcoding Supports “Color-Pattern’’-Based Species of Stictochironomus from China (Diptera: Chironomidae)
Source: Insects. 2024 Mar 6;15(3):179. doi: 10.3390/insects15030179 (PMC10971086; doi:10.3390/insects15030179)
Supplement: Supplementary file 1 [file insects-15-00179-s001.zip › insects-2882236-supplementary/S10.pdf]

ASAP Web results

date:2023-05-22T10:11:52

input file:hap-41.fas

nb of sequences:75

length of seqs:658

subst. model:K80 Kimura

recurs split pval:1.000000e-02

Split probability: 0.010000 sequence length: 658

Save Spart text File **here**

Save Spart XML File **here**

10 best partitions found by ASAP (see **FAQ** for more details)

The lower the score, the better the partition

Nb of subsets	asap-score		P-val (rank)	W (rank)	Treshold dist.	Text
20	3.00		5.87e-02 (1)	5.26e-04 (5)	0.076881	**list csv**
* 24	3.00		4.47e-01 (4)	4.50e-03 (2)	0.044953	**list csv**
19	5.00		2.14e-01 (3)	4.98e-04 (7)	0.078427	**list csv**
23	6.50		5.61e-01 (5)	4.61e-04 (8)	0.053311	**list csv**
21	8.00		8.52e-01 (12)	5.68e-04 (4)	0.068800	**list csv**
* 38	12.50		9.10e-01 (15)	4.60e-04 (10)	0.010322	**list csv**
* 36	12.50		9.42e-01 (16)	4.60e-04 (9)	0.010339	**list csv**
* 32	13.00		7.72e-01 (10)	3.15e-04 (16)	0.012984	**list csv**
15	13.50		1.30e-01 (2)	1.80e-04 (25)	0.090405	**list csv**
* 34	14.00		8.52e-01 (13)	3.15e-04 (15)	0.012921	**list csv**

Number of subsets in this table equals to the number of primary species hypotheses in the respective partition

Histogram of distances **[save]**

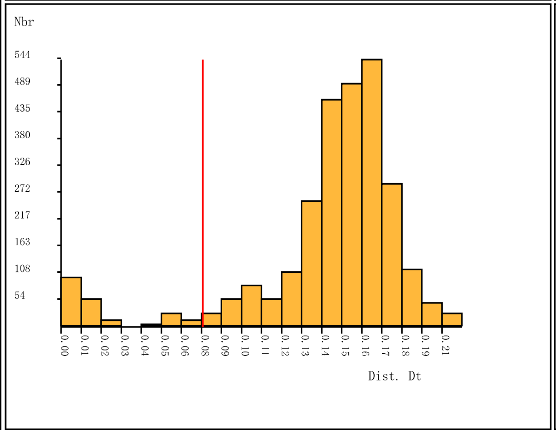

Ranked distances **[save]**

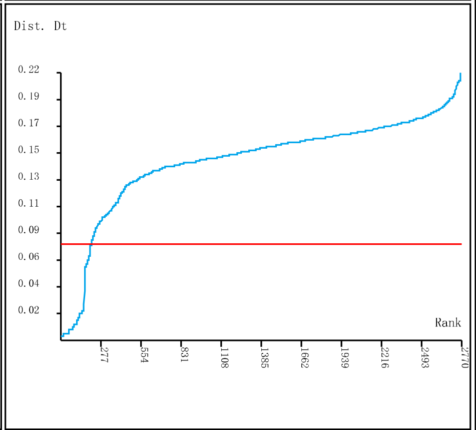

View/Save Boxed subsets graph **here**

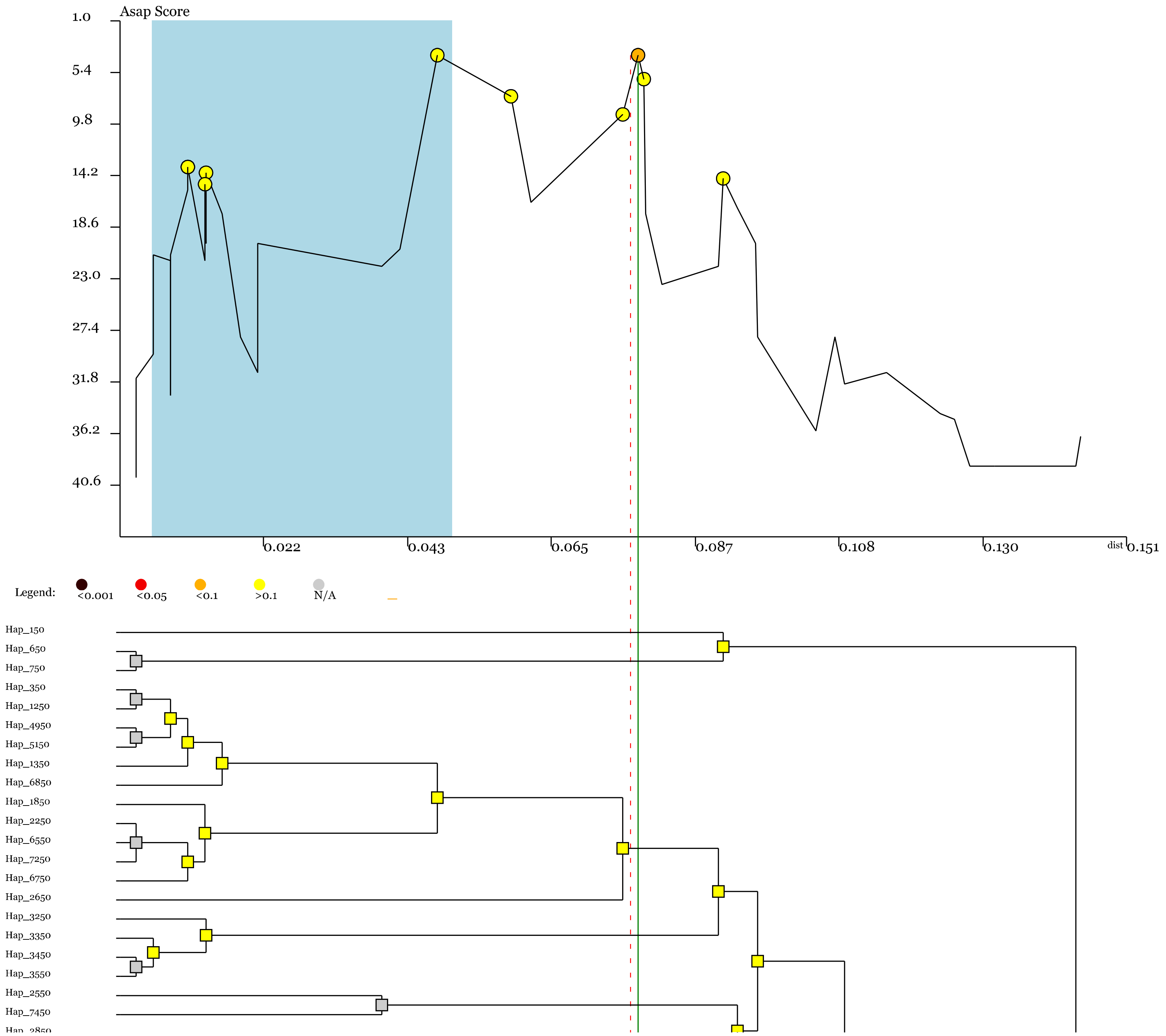
Asap Score
1.0
5.4
9.8
14.2
18.6
23.0
27.4
31.8
36.2
40.6
0.022
0.043
0.065
0.087
0.108
0.130
dist
0.151
Legend:
<0.001
<0.05
<0.1
>0.1
N/A
Hap_150
Hap_650
Hap_750
Hap_350
Hap_1250
Hap_4950
Hap_5150
Hap_1350
Hap_6850
Hap_1850
Hap_2250
Hap_6550
Hap_7250
Hap_6750
Hap_2650
Hap_3250
Hap_3350
Hap_3450
Hap_3550
Hap_2550
Hap_7450

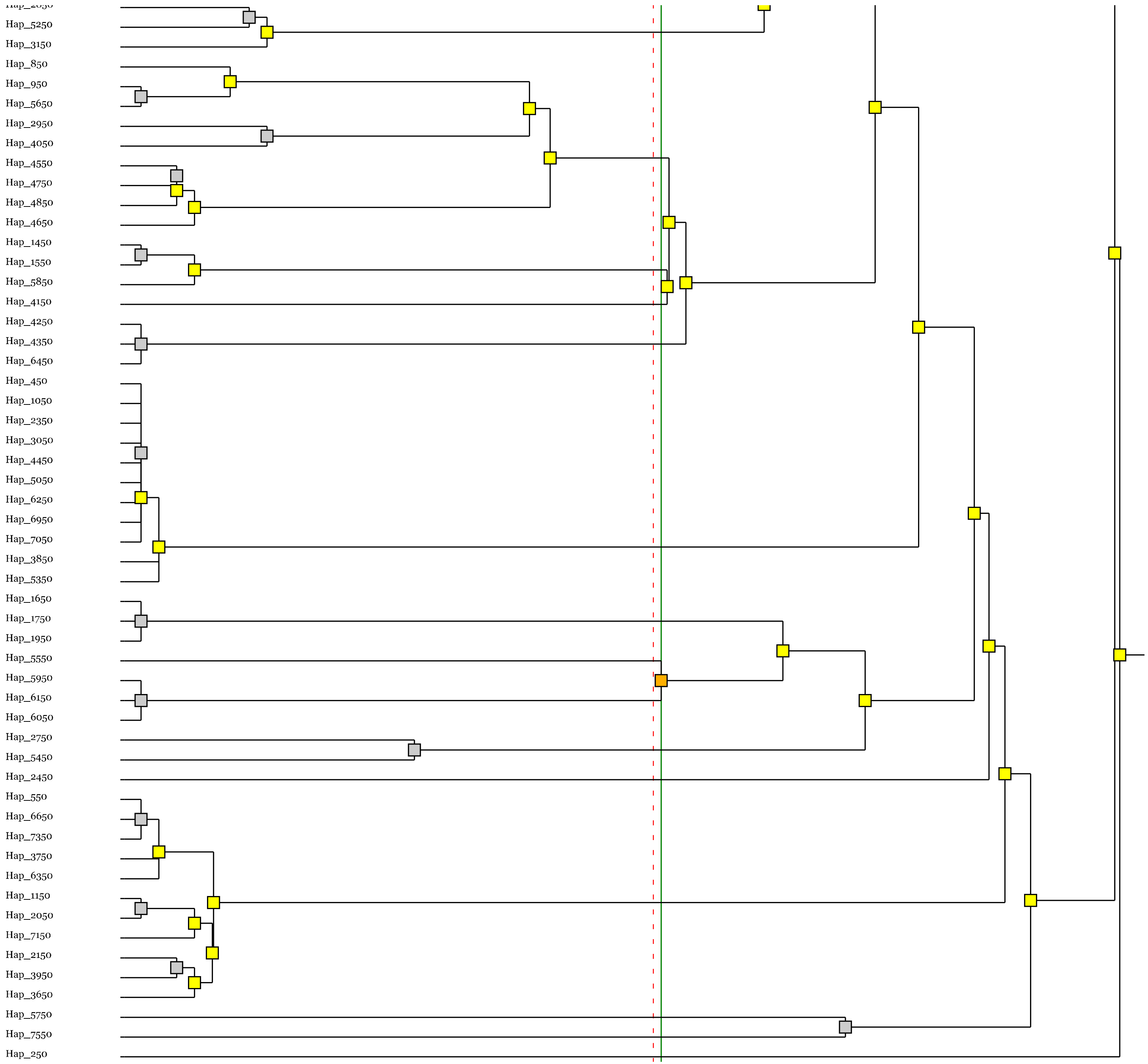
Hap_5250
Hap_3150
Hap_850
Hap_950
Hap_5650
Hap_2950
Hap_4050
Hap_4550
Hap_4750
Hap_4850
Hap_4650
Hap_1450
Hap_1550
Hap_5850
Hap_4150
Hap_4250
Hap_4350
Hap_6450
Hap_450
Hap_1050
Hap_2350
Hap_3050
Hap_4450
Hap_5050
Hap_6250
Hap_6950
Hap_7050
Hap_3850
Hap_5350
Hap_1650
Hap_1750
Hap_1950
Hap_5550
Hap_5950
Hap_6150
Hap_6050
Hap_2750
Hap_5450
Hap_2450
Hap_550
Hap_6650
Hap_7350
Hap_3750
Hap_6350
Hap_1150
Hap_2050
Hap_7150
Hap_2150
Hap_3950
Hap_3650
Hap_5750
Hap_7550
Hap_250

View/save curves and dendrogram **here**

Responsive Crossing lines Legend: Green Line= grouping distance (Dc)- Red line = treshold distance(Dt)

Running time: 0 min 9 seconds
