# Supplementary material for: DNA Barcoding Supports “Color-Pattern’’-Based Species of Stictochironomus from China (Diptera: Chironomidae)
Source: Insects. 2024 Mar 6;15(3):179. doi: 10.3390/insects15030179 (PMC10971086; doi:10.3390/insects15030179)
Supplement: Supplementary file 1 [file insects-15-00179-s001.zip › insects-2882236-supplementary/S5.pdf]

ASAP Web results

date:2023-02-14T10:21:42

input file:Stictochironomus-Alignment-3.fas

nb of sequences:704

length of seqs:658

subst. model:K80 Kimura

recurs split pval:1.000000e-02

Split probability: 0.010000 sequence length: 658

Save Spart text File **here**

Save Spart XML File **here**

10 best partitions found by ASAP (see **FAQ** for more details)

The lower the score, the better the partition

Nb of subsets	asap-score	P-val (rank)	W (rank)	Treshold dist.	Text
* 26	2.50	8.55e-03 (2)	9.71e-04 (3)	0.048527	**list csv**
* 27	3.50	2.46e-02 (5)	1.87e-03 (2)	0.026290	**list csv**
24	4.50	2.01e-02 (4)	7.64e-05 (5)	0.069020	**list csv**
22	8.50	1.70e-02 (3)	6.09e-05 (14)	0.073653	**list csv**
* 45	10.50	4.79e-02 (9)	6.27e-05 (12)	0.006204	**list csv**
19	10.50	6.32e-02 (11)	6.55e-05 (10)	0.088886	**list csv**
26	12.00	2.26e-01 (20)	2.16e-04 (4)	0.056231	**list csv**
* 38	17.00	2.57e-02 (6)	2.63e-05 (28)	0.009415	**list csv**
13	18.50	1.00e-05 (1)	7.11e-06 (36)	0.104006	**list csv**
15	20.50	4.01e-02 (8)	1.83e-05 (33)	0.101450	**list csv**

Number of subsets in this table equals to the number of primary species hypotheses in the respective partition

Histogram of distances **[save]**

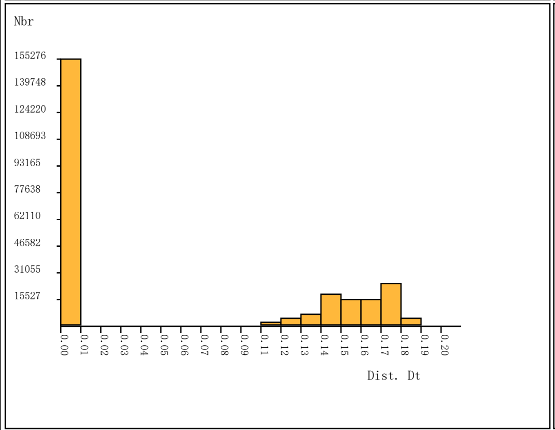

Ranked distances **[save]**

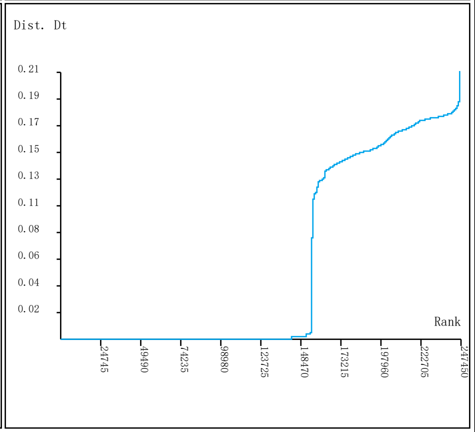

View/Save Boxed subsets graph **here**

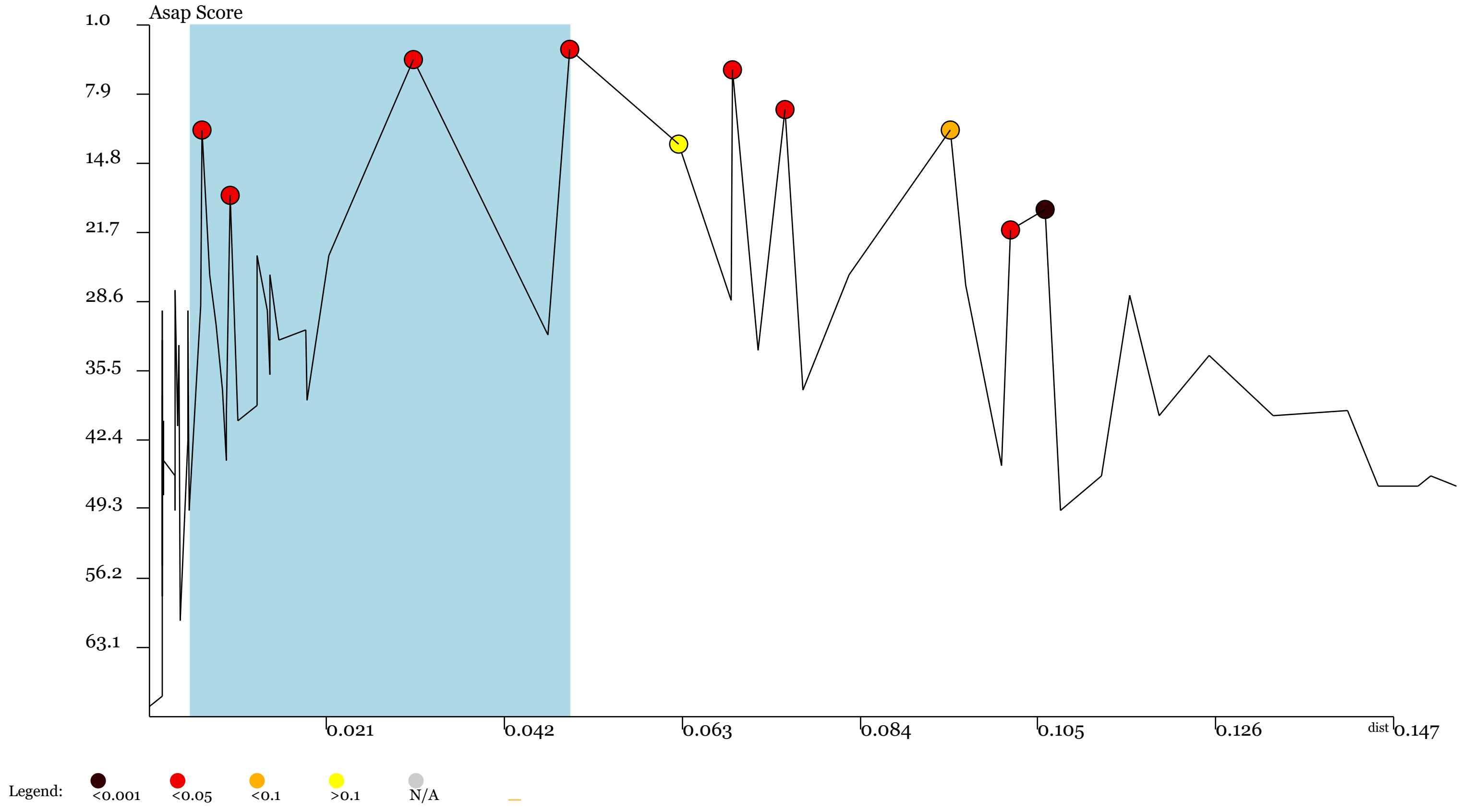
Asap Score
1.0
7.9
14.8
21.7
28.6
35.5
42.4
49.3
56.2
63.1
0.021
0.042
0.063
0.084
0.105
0.126
dist
0.147
Legend:
<0.001
<0.05
<0.1
>0.1
N/A

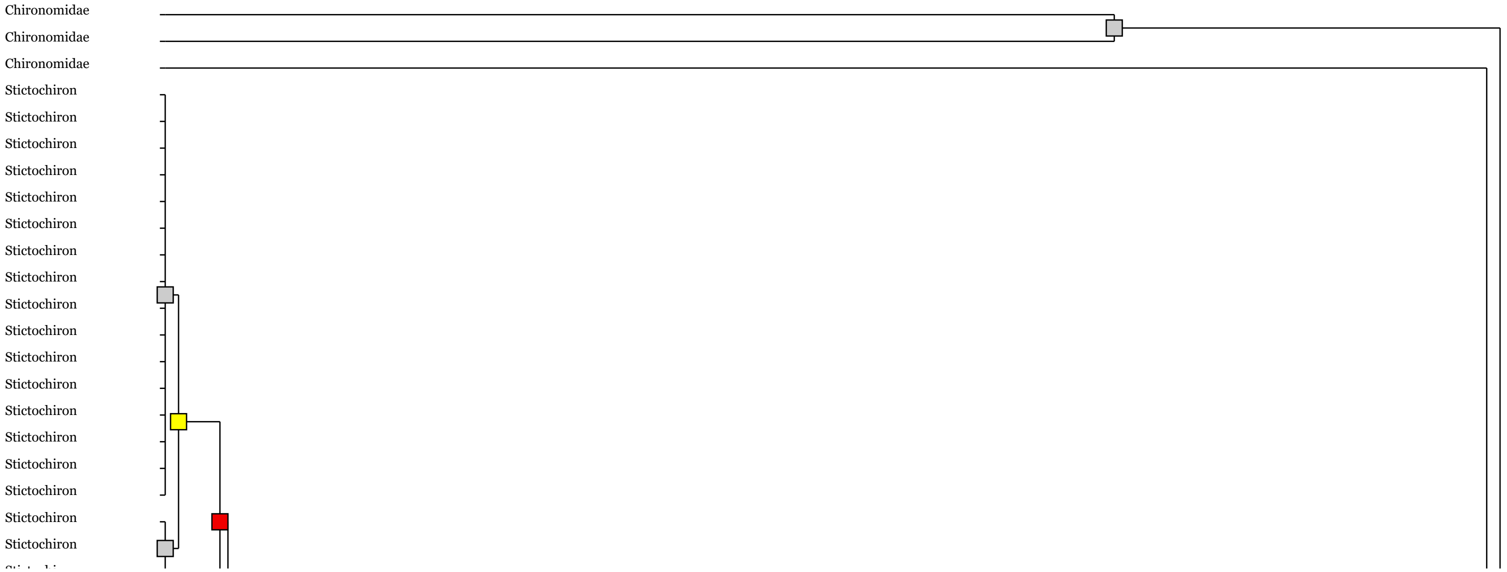
Chironomidae
Chironomidae
Chironomidae
Stictochiron
Stictochiron
Stictochiron
Stictochiron
Stictochiron
Stictochiron
Stictochiron
Stictochiron
Stictochiron
Stictochiron
Stictochiron
Stictochiron
Stictochiron
Stictochiron
Stictochiron
Stictochiron
Stictochiron
Stictochiron

View/save curves and dendrogram **here**

Responsive Crossing lines Legend: Green Line= grouping distance (Dc)- Red line = treshold distance(Dt)

Running time: 0 min 30 seconds

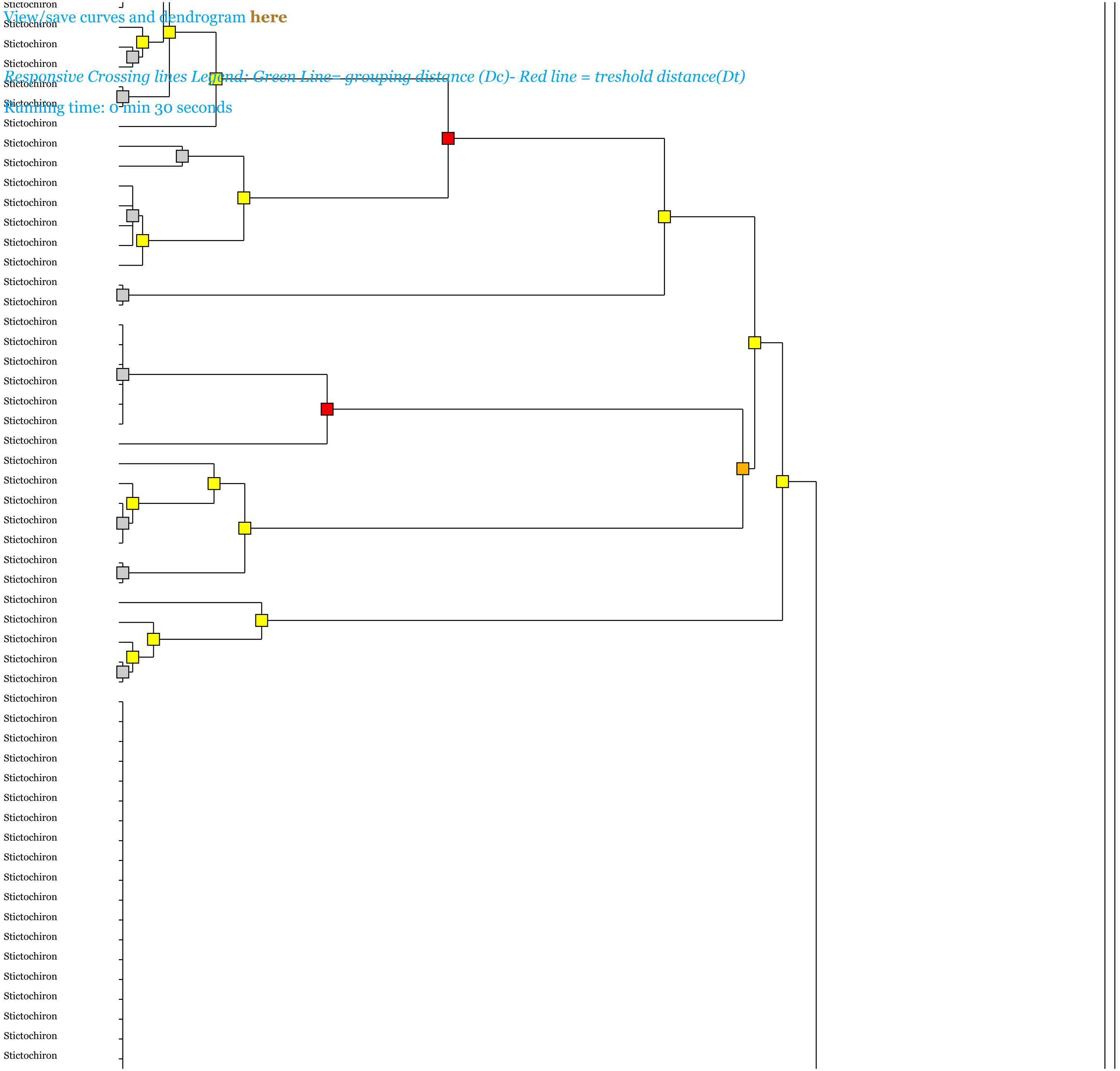

Stictochiron
Stictochiron
Stictochiron
Stictochiron
Stictochiron
Stictochiron
Stictochiron
Stictochiron
Stictochiron
Stictochiron
Stictochiron
Stictochiron
Stictochiron
Stictochiron
Stictochiron
Stictochiron
Stictochiron
Stictochiron
Stictochiron
Stictochiron
Stictochiron
Stictochiron
Stictochiron
Stictochiron
Stictochiron
Stictochiron
Stictochiron
Stictochiron
Stictochiron
Stictochiron
Stictochiron
Stictochiron
Stictochiron
Stictochiron
Stictochiron
Stictochiron
Stictochiron
Stictochiron
Stictochiron
Stictochiron
Stictochiron
Stictochiron
Stictochiron
Stictochiron
Stictochiron
Stictochiron
Stictochiron
Stictochiron
Stictochiron
Stictochiron
Stictochiron
Stictochiron
Stictochiron
Stictochiron

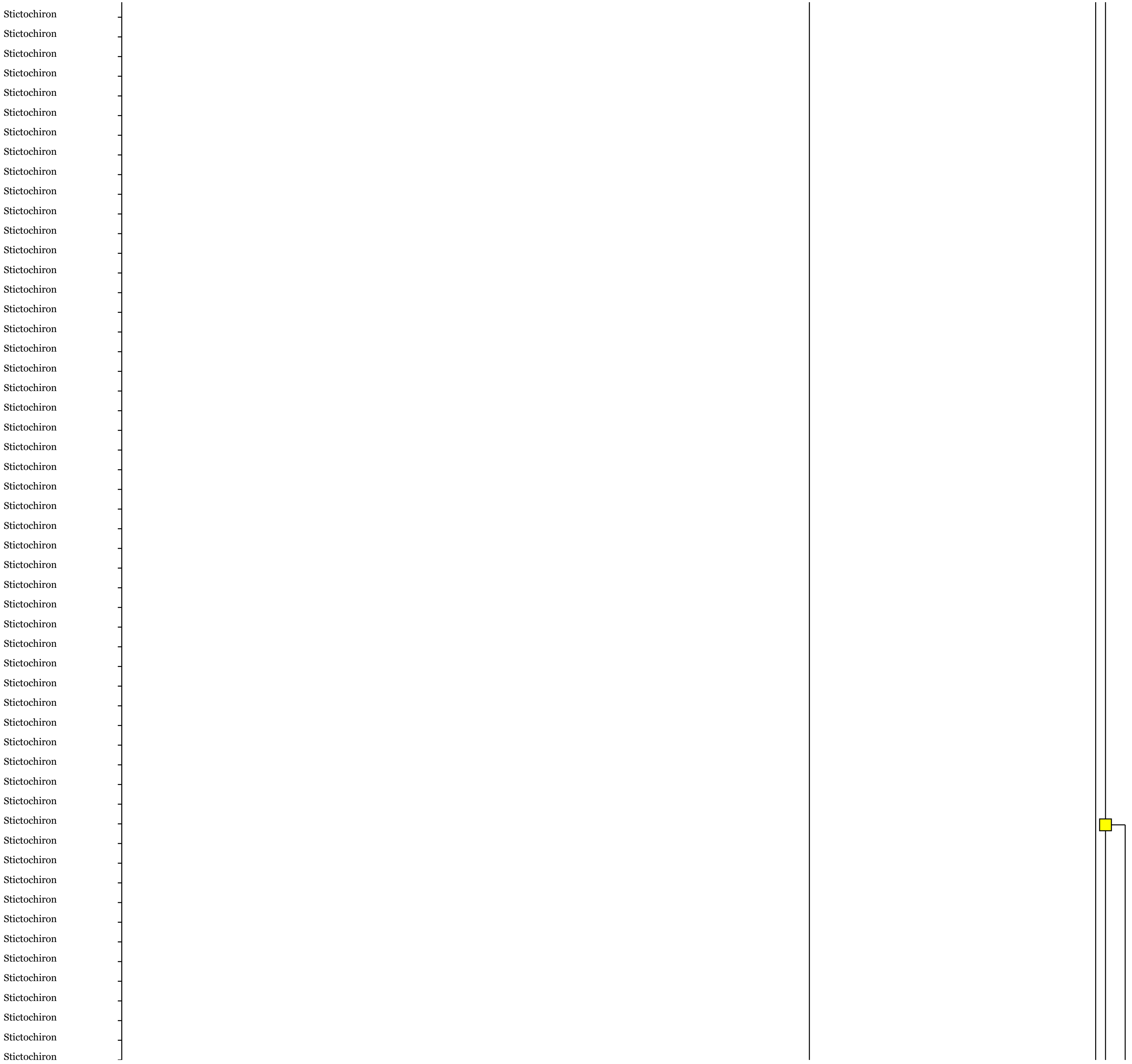
Stictochiron
Stictochiron
Stictochiron
Stictochiron
Stictochiron
Stictochiron
Stictochiron
Stictochiron
Stictochiron
Stictochiron
Stictochiron
Stictochiron
Stictochiron
Stictochiron
Stictochiron
Stictochiron
Stictochiron
Stictochiron
Stictochiron
Stictochiron
Stictochiron
Stictochiron
Stictochiron
Stictochiron
Stictochiron
Stictochiron
Stictochiron
Stictochiron
Stictochiron
Stictochiron
Stictochiron
Stictochiron
Stictochiron
Stictochiron
Stictochiron
Stictochiron
Stictochiron
Stictochiron
Stictochiron
Stictochiron
Stictochiron
Stictochiron
Stictochiron
Stictochiron
Stictochiron
Stictochiron
Stictochiron
Stictochiron
Stictochiron
Stictochiron
Stictochiron
Stictochiron
Stictochiron
Stictochiron

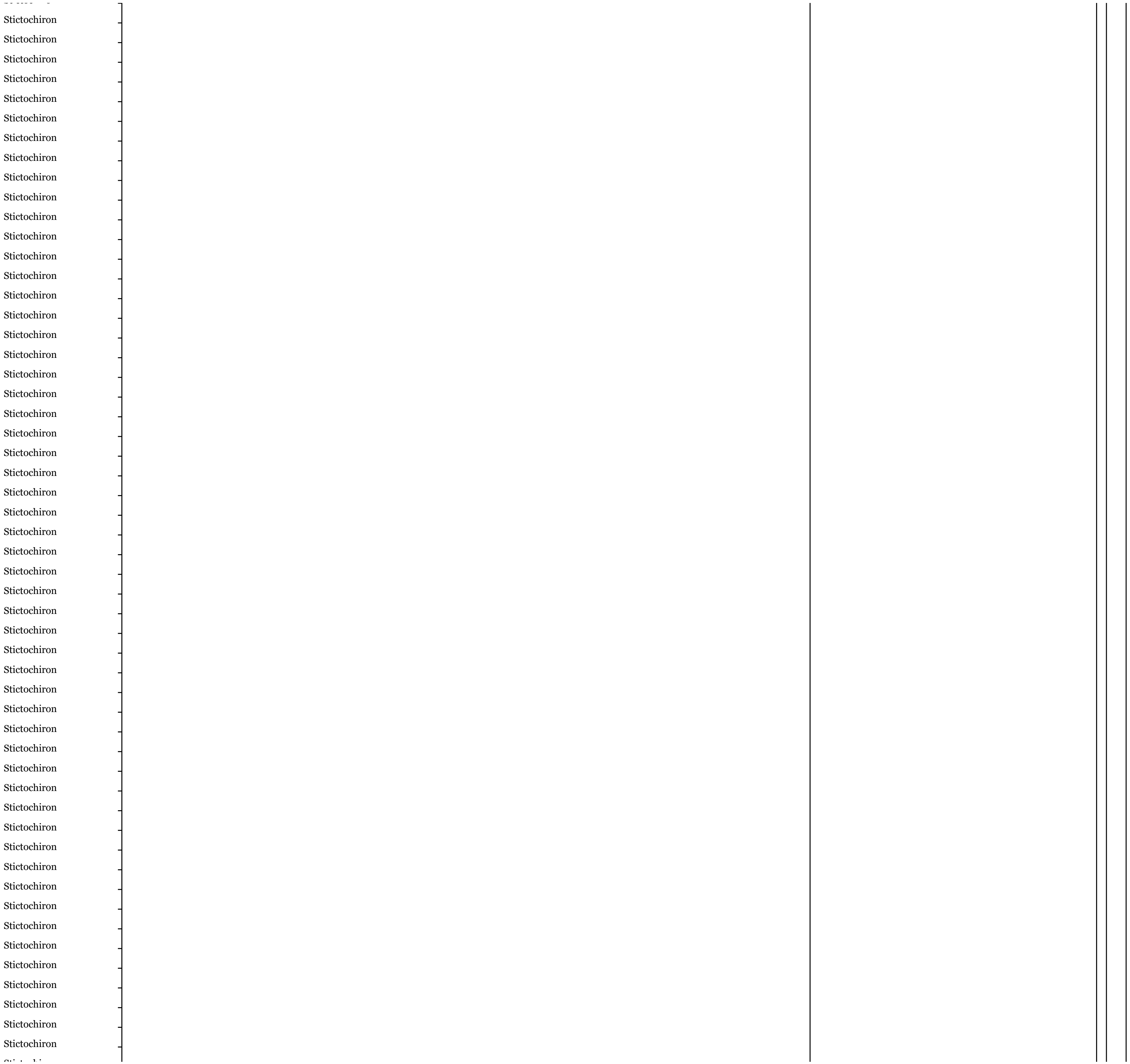
Stictochiron
Stictochiron
Stictochiron
Stictochiron
Stictochiron
Stictochiron
Stictochiron
Stictochiron
Stictochiron
Stictochiron
Stictochiron
Stictochiron
Stictochiron
Stictochiron
Stictochiron
Stictochiron
Stictochiron
Stictochiron
Stictochiron
Stictochiron
Stictochiron
Stictochiron
Stictochiron
Stictochiron
Stictochiron
Stictochiron
Stictochiron
Stictochiron
Stictochiron
Stictochiron
Stictochiron
Stictochiron
Stictochiron
Stictochiron
Stictochiron
Stictochiron
Stictochiron
Stictochiron
Stictochiron
Stictochiron
Stictochiron
Stictochiron
Stictochiron
Stictochiron
Stictochiron
Stictochiron
Stictochiron
Stictochiron
Stictochiron
Stictochiron
Stictochiron
Stictochiron
Stictochiron

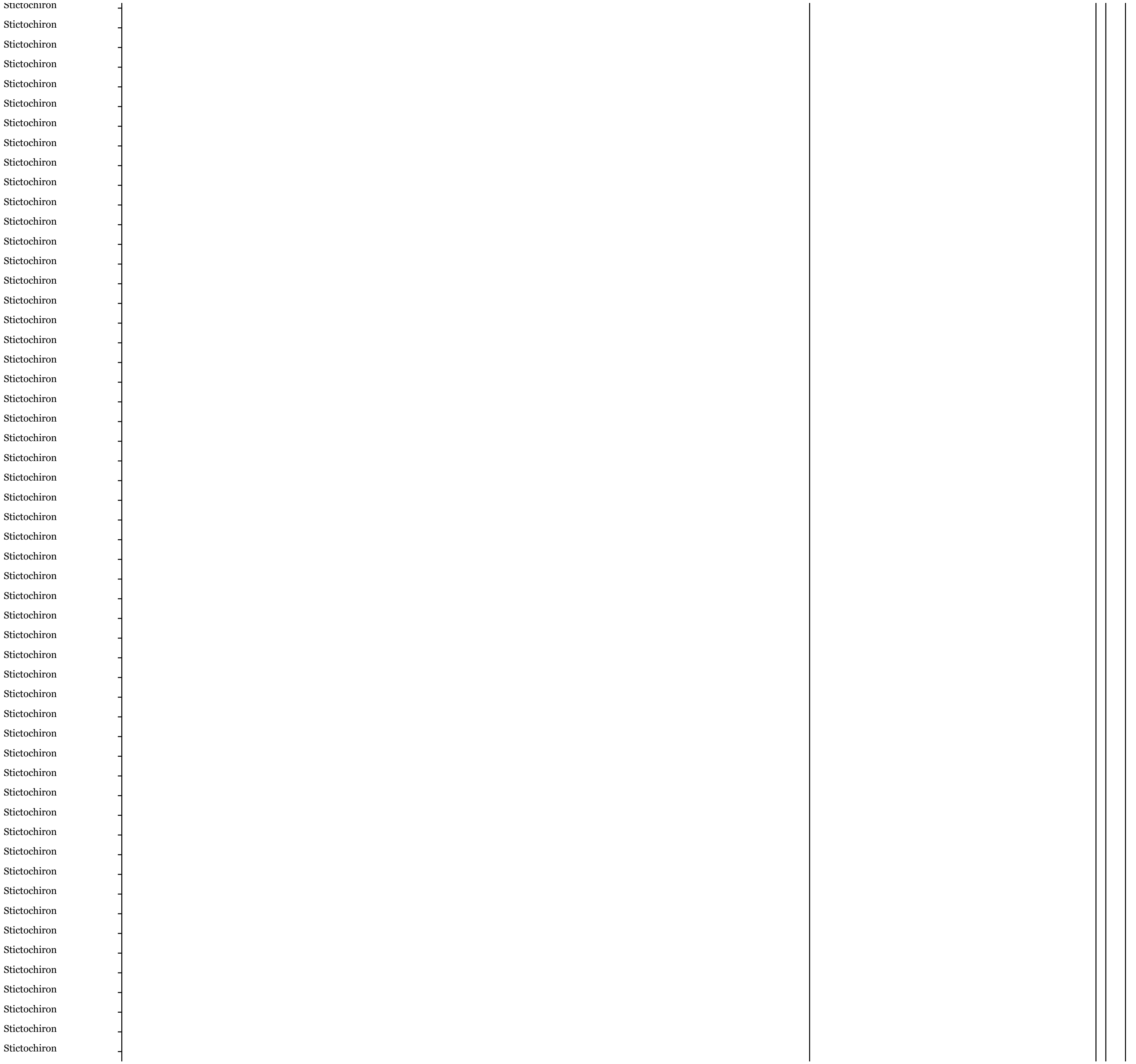
Stictochiron
Stictochiron
Stictochiron
Stictochiron
Stictochiron
Stictochiron
Stictochiron
Stictochiron
Stictochiron
Stictochiron
Stictochiron
Stictochiron
Stictochiron
Stictochiron
Stictochiron
Stictochiron
Stictochiron
Stictochiron
Stictochiron
Stictochiron
Stictochiron
Stictochiron
Stictochiron
Stictochiron
Stictochiron
Stictochiron
Stictochiron
Stictochiron
Stictochiron
Stictochiron
Stictochiron
Stictochiron
Stictochiron
Stictochiron
Stictochiron
Stictochiron
Stictochiron
Stictochiron
Stictochiron
Stictochiron
Stictochiron
Stictochiron
Stictochiron
Stictochiron
Stictochiron
Stictochiron
Stictochiron
Stictochiron
Stictochiron
Stictochiron
Stictochiron
Stictochiron
Stictochiron
Stictochiron

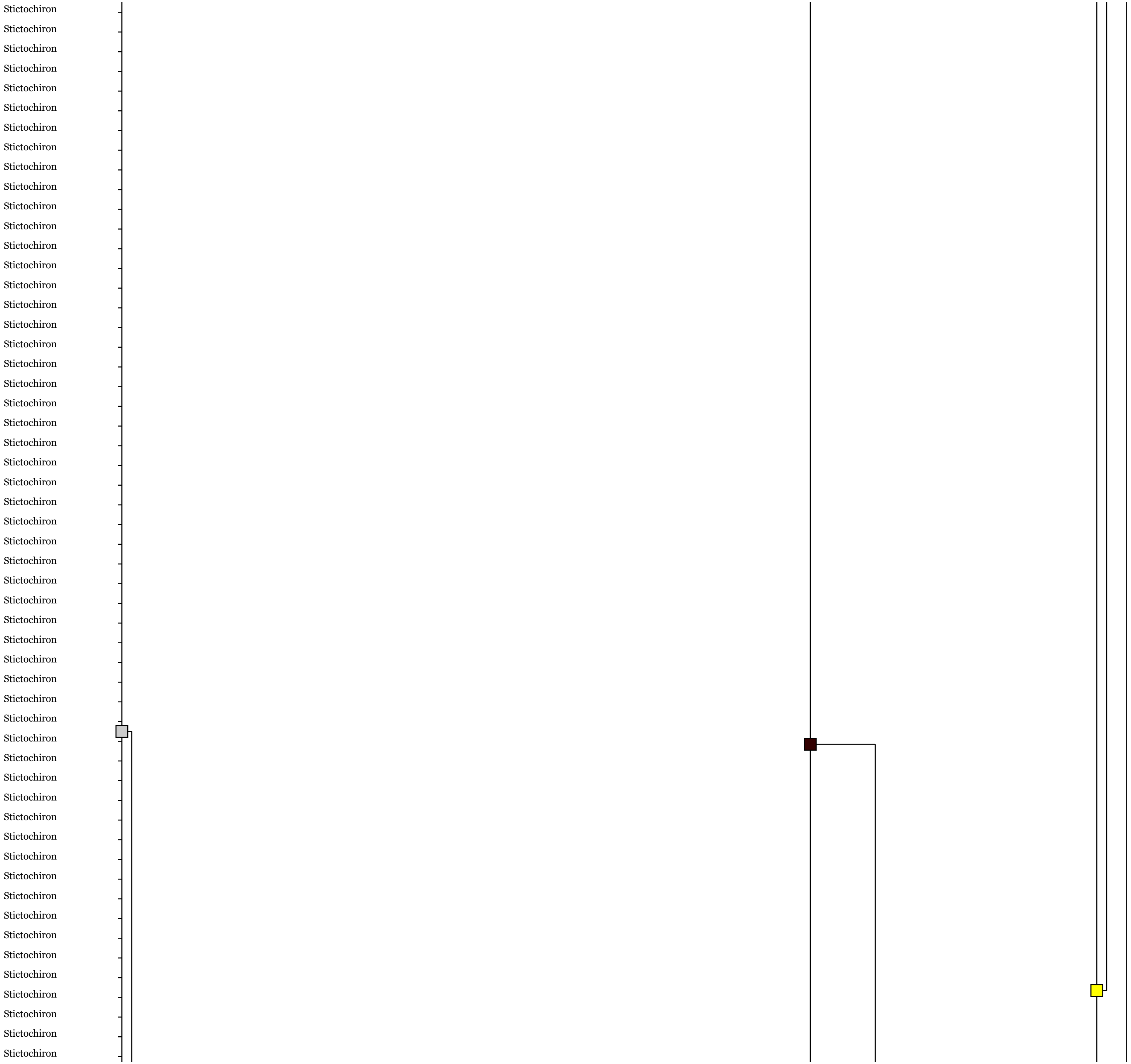
Stictochiron
Stictochiron
Stictochiron
Stictochiron
Stictochiron
Stictochiron
Stictochiron
Stictochiron
Stictochiron
Stictochiron
Stictochiron
Stictochiron
Stictochiron
Stictochiron
Stictochiron
Stictochiron
Stictochiron
Stictochiron
Stictochiron
Stictochiron
Stictochiron
Stictochiron
Stictochiron
Stictochiron
Stictochiron
Stictochiron
Stictochiron
Stictochiron
Stictochiron
Stictochiron
Stictochiron
Stictochiron
Stictochiron
Stictochiron
Stictochiron
Stictochiron
Stictochiron
Stictochiron
Stictochiron
Stictochiron
Stictochiron
Stictochiron
Stictochiron
Stictochiron
Stictochiron
Stictochiron
Stictochiron
Stictochiron
Stictochiron
Stictochiron
Stictochiron
Stictochiron
Stictochiron
Stictochiron

Stictochiron
Stictochiron
Stictochiron
Stictochiron
Stictochiron
Stictochiron
Stictochiron
Stictochiron
Stictochiron
Stictochiron
Stictochiron
Stictochiron
Stictochiron
Stictochiron
Stictochiron
Stictochiron
Stictochiron
Stictochiron
Stictochiron
Stictochiron
Stictochiron
Stictochiron
Stictochiron
Stictochiron
Stictochiron
Stictochiron
Stictochiron
Stictochiron
Stictochiron
Stictochiron
Stictochiron
Stictochiron
Stictochiron
Stictochiron
Stictochiron
Stictochiron
Stictochiron
Stictochiron
Stictochiron
Stictochiron
Stictochiron
Stictochiron
Stictochiron
Stictochiron
Stictochiron
Stictochiron
Stictochiron
Stictochiron
Stictochiron
Stictochiron
Stictochiron
Stictochiron
Stictochiron
Stictochiron

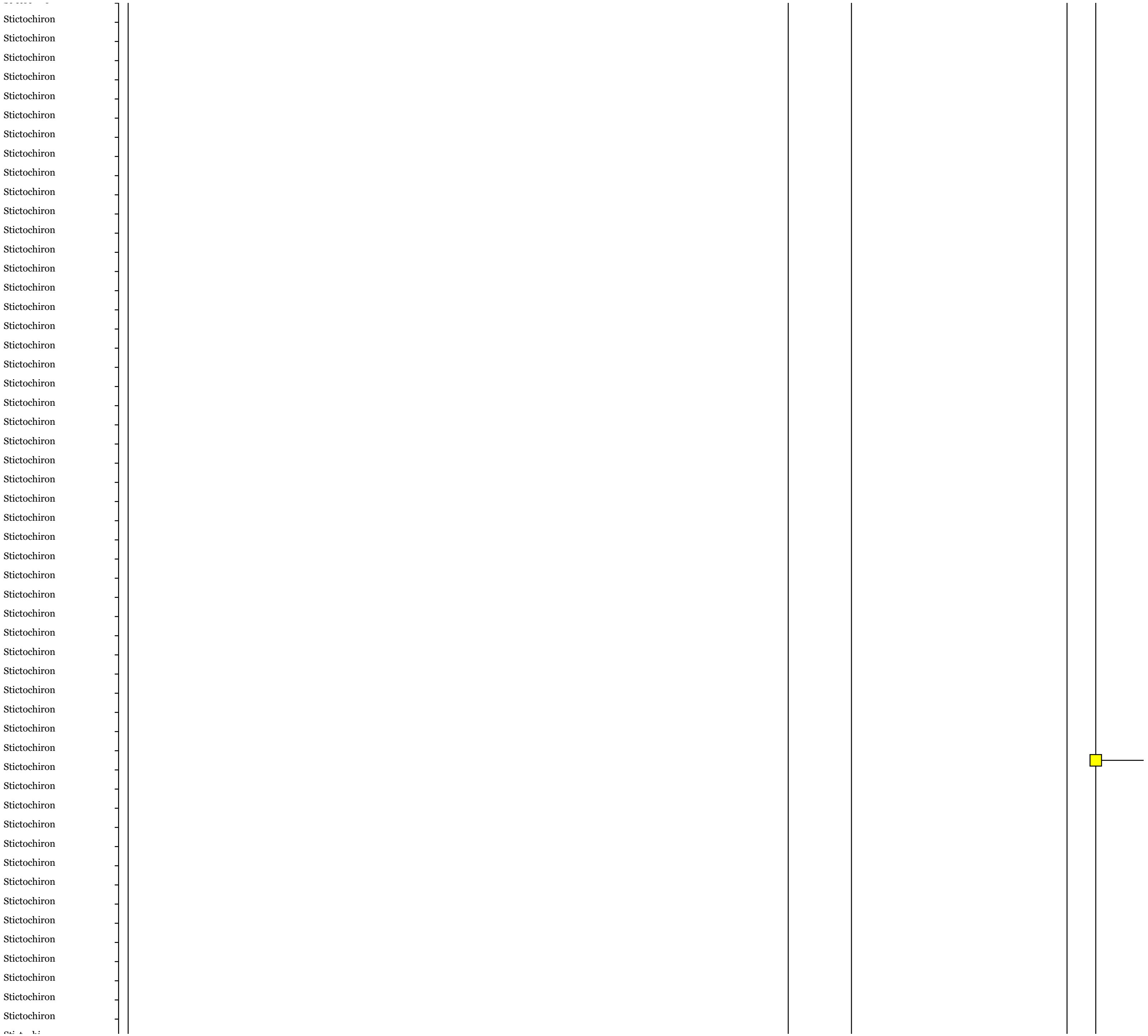
Stictochiron
Stictochiron
Stictochiron
Stictochiron
Stictochiron
Stictochiron
Stictochiron
Stictochiron
Stictochiron
Stictochiron
Stictochiron
Stictochiron
Stictochiron
Stictochiron
Stictochiron
Stictochiron
Stictochiron
Stictochiron
Stictochiron
Stictochiron
Stictochiron
Stictochiron
Stictochiron
Stictochiron
Stictochiron
Stictochiron
Stictochiron
Stictochiron
Stictochiron
Stictochiron
Stictochiron
Stictochiron
Stictochiron
Stictochiron
Stictochiron
Stictochiron
Stictochiron
Stictochiron
Stictochiron
Stictochiron
Stictochiron
Stictochiron
Stictochiron
Stictochiron
Stictochiron
Stictochiron
Stictochiron
Stictochiron
Stictochiron
Stictochiron
Stictochiron
Stictochiron
Stictochiron

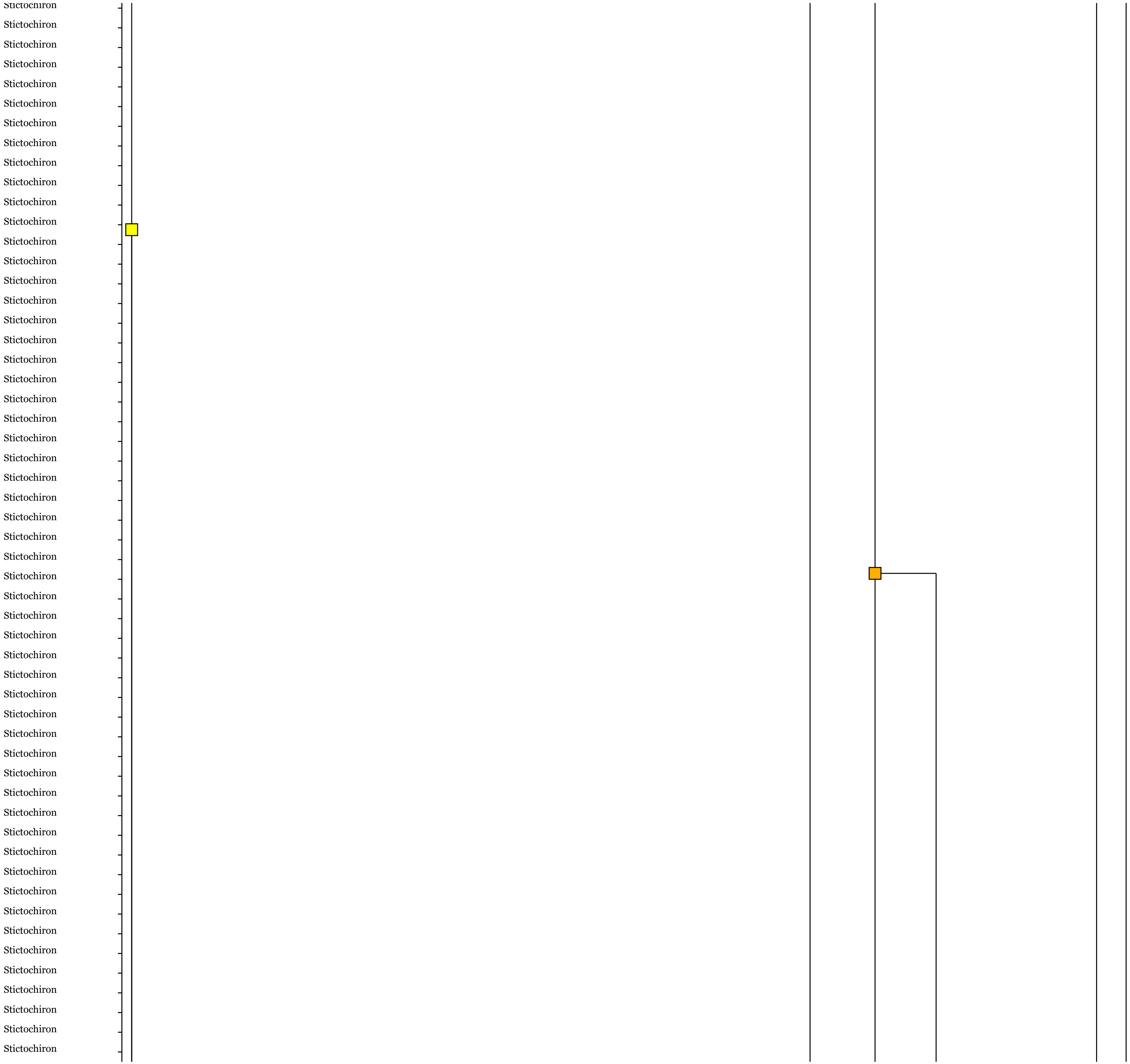
Stictochiron
Stictochiron
Stictochiron
Stictochiron
Stictochiron
Stictochiron
Stictochiron
Stictochiron
Stictochiron
Stictochiron
Stictochiron
Stictochiron
Stictochiron
Stictochiron
Stictochiron
Stictochiron
Stictochiron
Stictochiron
Stictochiron
Stictochiron
Stictochiron
Stictochiron
Stictochiron
Stictochiron
Stictochiron
Stictochiron
Stictochiron
Stictochiron
Stictochiron
Stictochiron
Stictochiron
Stictochiron
Stictochiron
Stictochiron
Stictochiron
Stictochiron
Stictochiron
Stictochiron
Stictochiron
Stictochiron
Stictochiron
Stictochiron
Stictochiron
Stictochiron
Stictochiron
Stictochiron
Stictochiron
Stictochiron
Stictochiron
Stictochiron
Stictochiron
Stictochiron
Stictochiron
Stictochiron

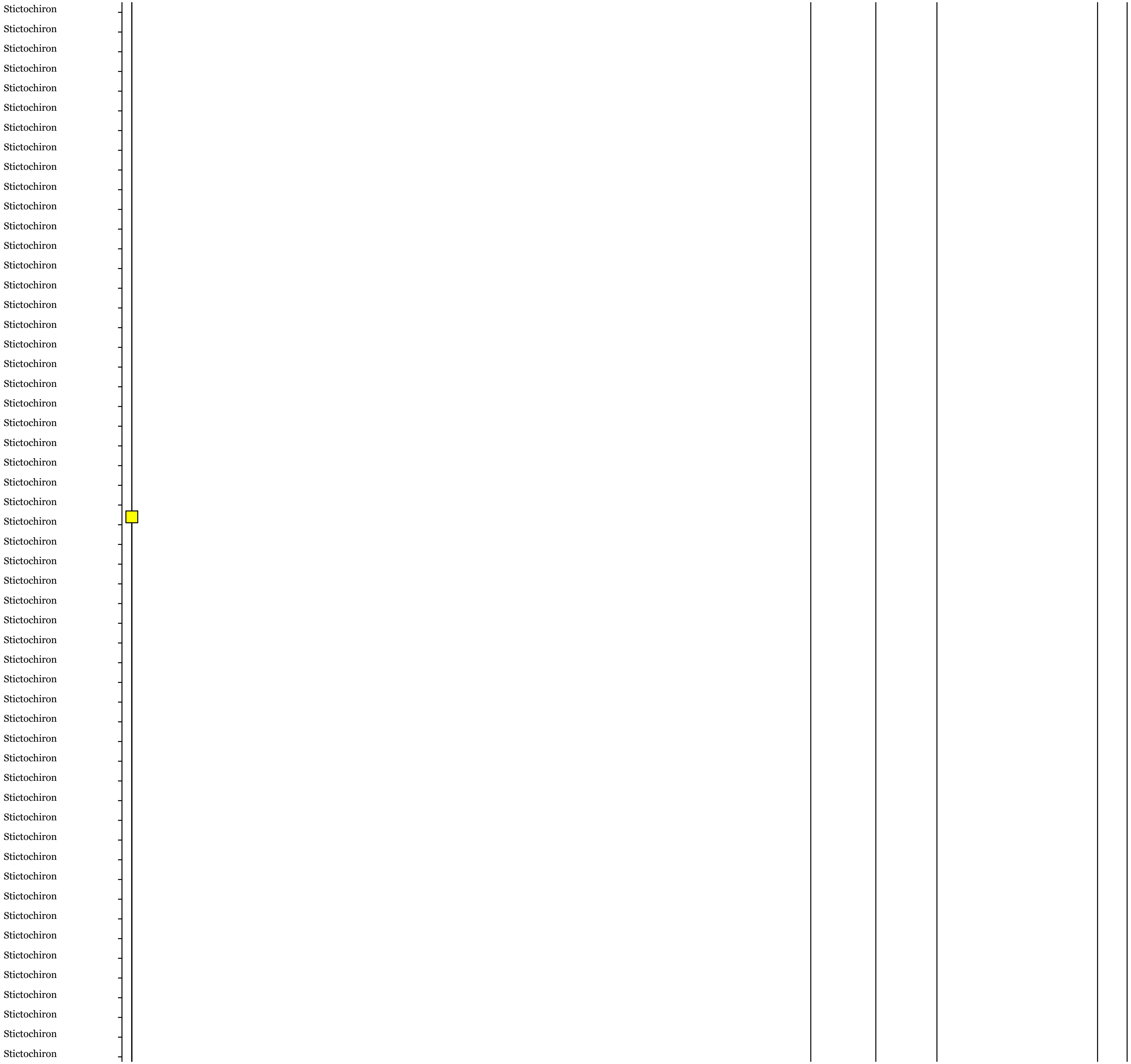
Stictochiron
Stictochiron
Stictochiron
Stictochiron
Stictochiron
Stictochiron
Stictochiron
Stictochiron
Stictochiron
Stictochiron
Stictochiron
Stictochiron
Stictochiron
Stictochiron
Stictochiron
Stictochiron
Stictochiron
Stictochiron
Stictochiron
Stictochiron
Stictochiron
Stictochiron
Stictochiron
Stictochiron
Stictochiron
Stictochiron
Stictochiron
Stictochiron
Stictochiron
Stictochiron
Stictochiron
Stictochiron
Stictochiron
Stictochiron
Stictochiron
Stictochiron
Stictochiron
Stictochiron
Stictochiron
Stictochiron
Stictochiron
Stictochiron
Stictochiron
Stictochiron
Stictochiron
Stictochiron
Stictochiron
Stictochiron
Stictochiron
Stictochiron
Stictochiron
Stictochiron
Stictochiron
Stictochiron

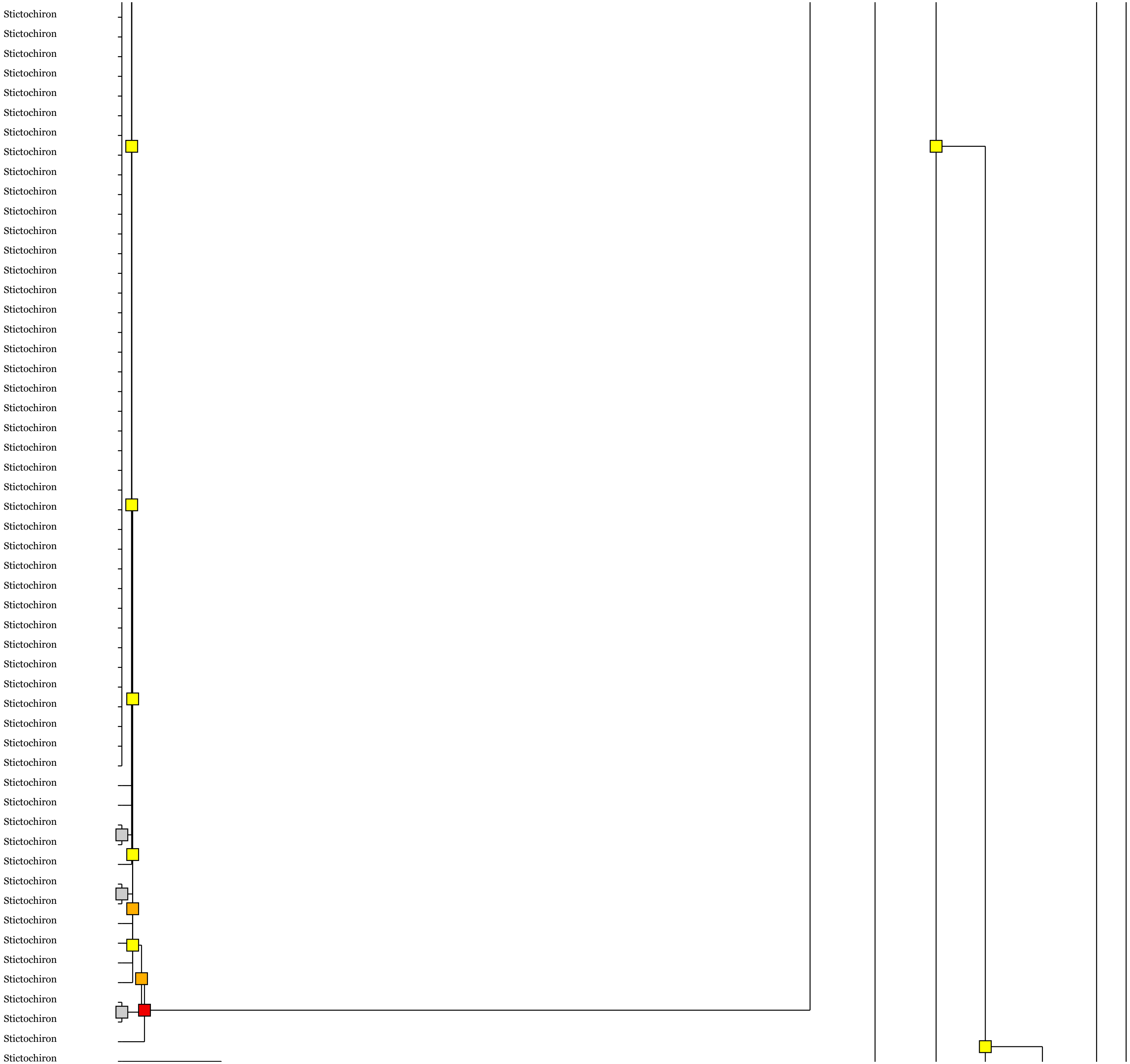
Stictochiron
Stictochiron
Stictochiron
Stictochiron
Stictochiron
Stictochiron
Stictochiron
Stictochiron
Stictochiron
Stictochiron
Stictochiron
Stictochiron
Stictochiron
Stictochiron
Stictochiron
Stictochiron
Stictochiron
Stictochiron
Stictochiron
Stictochiron
Stictochiron
Stictochiron
Stictochiron
Stictochiron
Stictochiron
Stictochiron
Stictochiron
Stictochiron
Stictochiron
Stictochiron
Stictochiron
Stictochiron
Stictochiron
Stictochiron
Stictochiron
Stictochiron
Stictochiron
Stictochiron
Stictochiron
Stictochiron
Stictochiron
Stictochiron
Stictochiron
Stictochiron
Stictochiron
Stictochiron
Stictochiron
Stictochiron
Stictochiron
Stictochiron
Stictochiron
Stictochiron
Stictochiron
Stictochiron

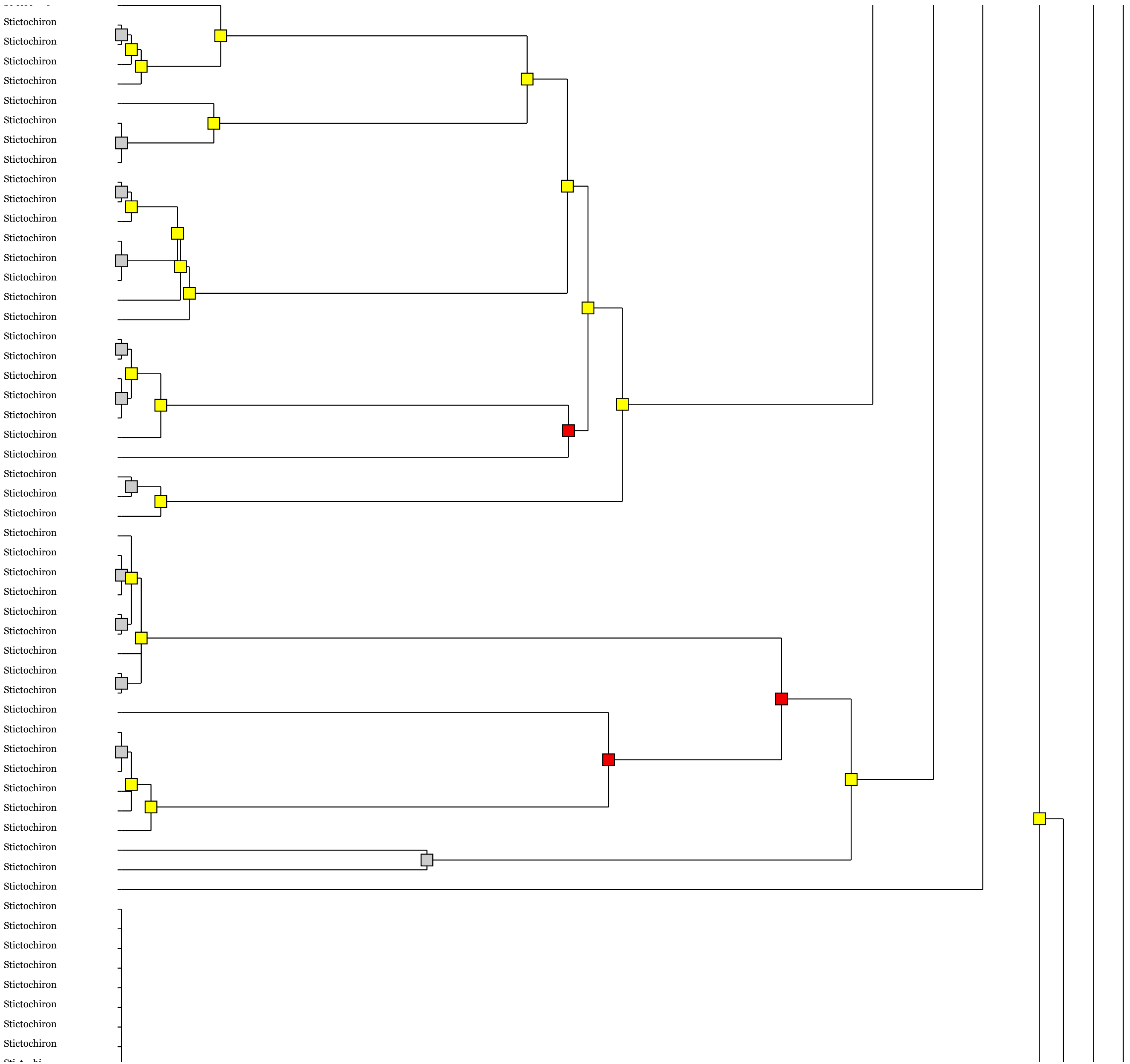
Stictochiron
Stictochiron
Stictochiron
Stictochiron
Stictochiron
Stictochiron
Stictochiron
Stictochiron
Stictochiron
Stictochiron
Stictochiron
Stictochiron
Stictochiron
Stictochiron
Stictochiron
Stictochiron
Stictochiron
Stictochiron
Stictochiron
Stictochiron
Stictochiron
Stictochiron
Stictochiron
Stictochiron
Stictochiron
Stictochiron
Stictochiron
Stictochiron
Stictochiron
Stictochiron
Stictochiron
Stictochiron
Stictochiron
Stictochiron
Stictochiron
Stictochiron
Stictochiron
Stictochiron
Stictochiron
Stictochiron
Stictochiron
Stictochiron
Stictochiron
Stictochiron
Stictochiron
Stictochiron
Stictochiron
Stictochiron
Stictochiron
Stictochiron
Stictochiron
Stictochiron
Stictochiron

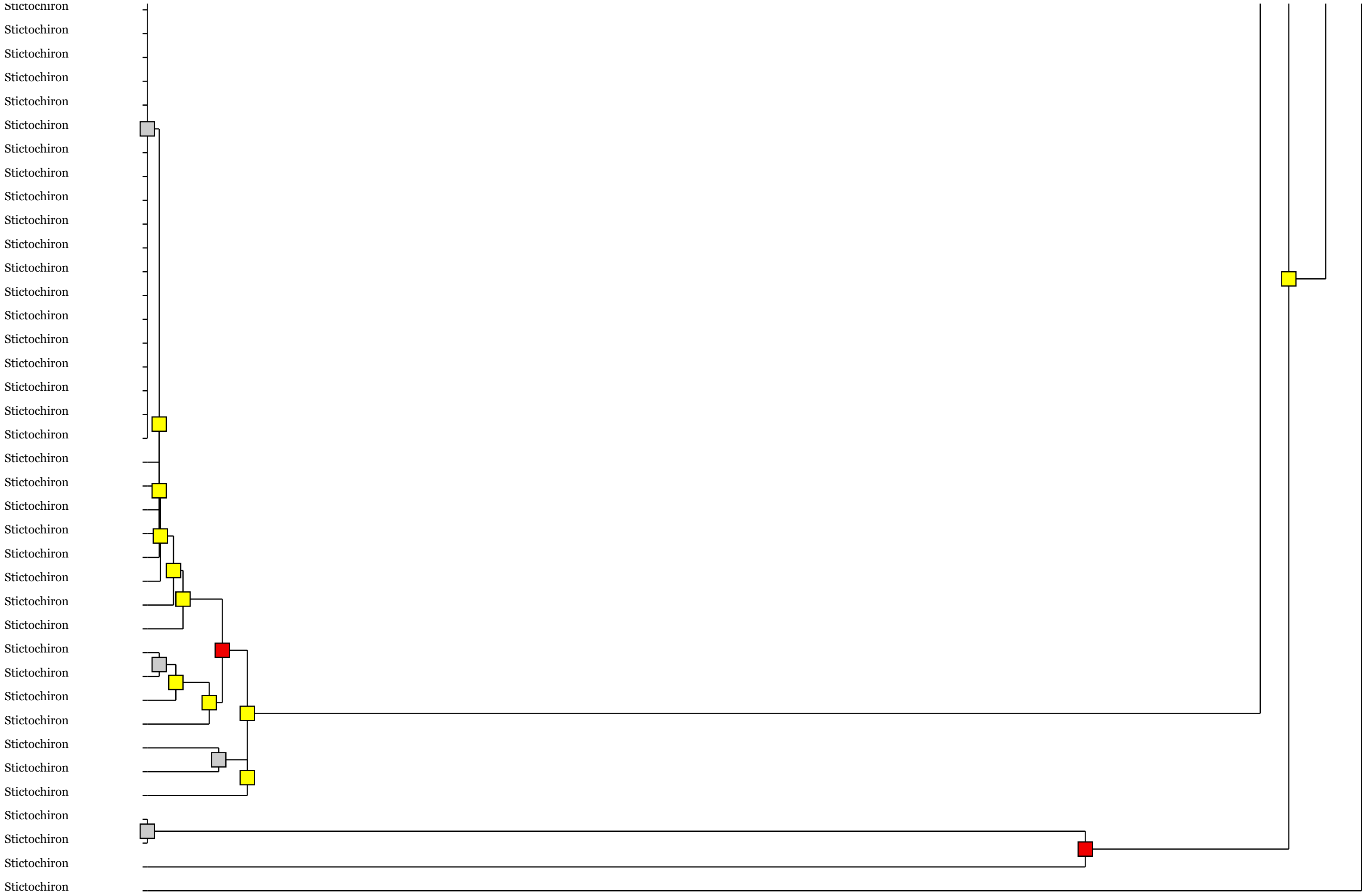
Stictochiron
Stictochiron
Stictochiron
Stictochiron
Stictochiron
Stictochiron
Stictochiron
Stictochiron
Stictochiron
Stictochiron
Stictochiron
Stictochiron
Stictochiron
Stictochiron
Stictochiron
Stictochiron
Stictochiron
Stictochiron
Stictochiron
Stictochiron
Stictochiron
Stictochiron
Stictochiron
Stictochiron
Stictochiron
Stictochiron
Stictochiron
Stictochiron
Stictochiron
Stictochiron
Stictochiron
Stictochiron
Stictochiron
Stictochiron
Stictochiron
Stictochiron
Stictochiron
Stictochiron
