# Supplementary figures and images for: DNA Barcoding Supports “Color-Pattern’’-Based Species of Stictochironomus from China (Diptera: Chironomidae)
Source: Insects. 2024 Mar 6;15(3):179. doi: 10.3390/insects15030179 (PMC10971086; doi:10.3390/insects15030179)

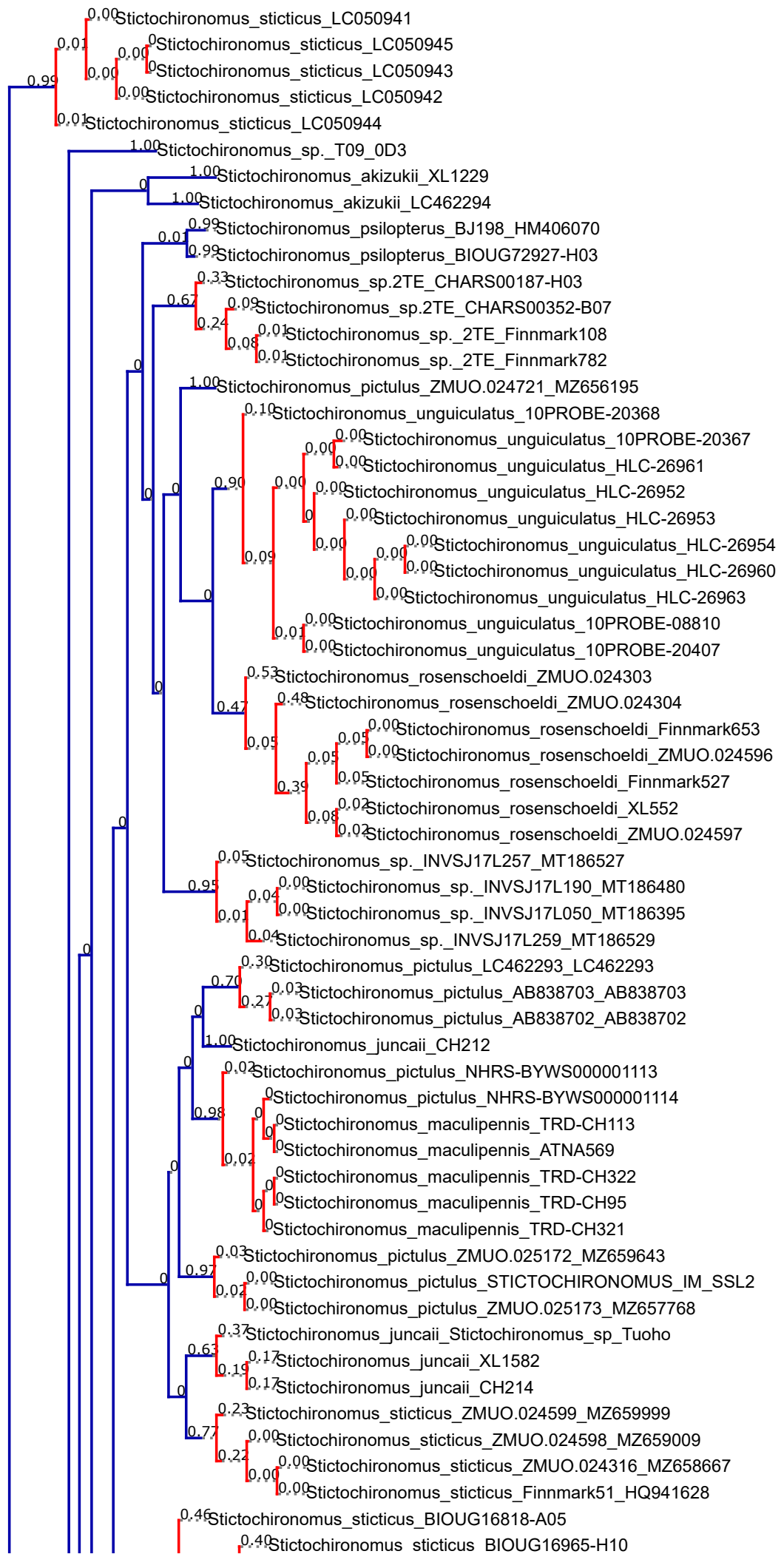

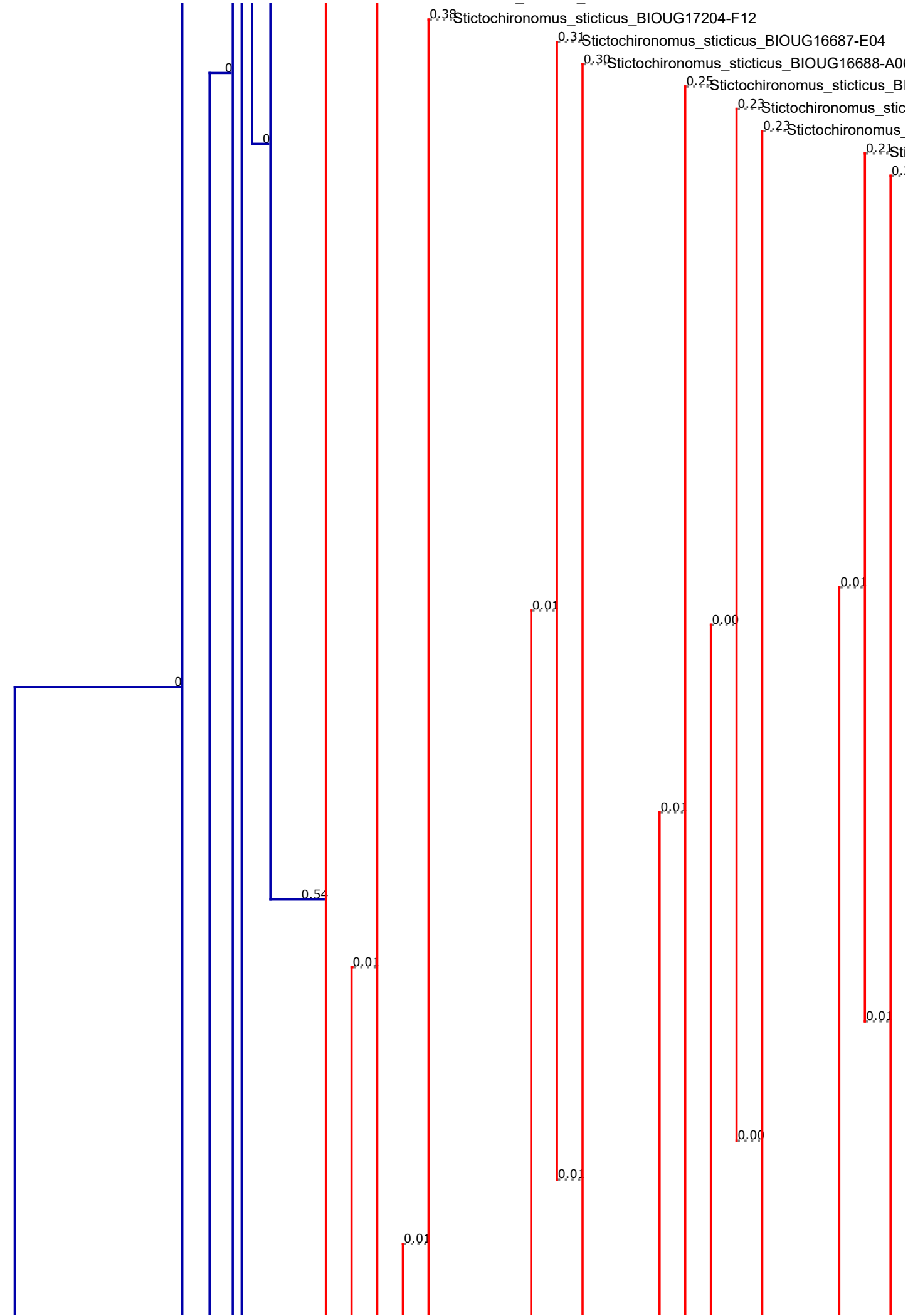

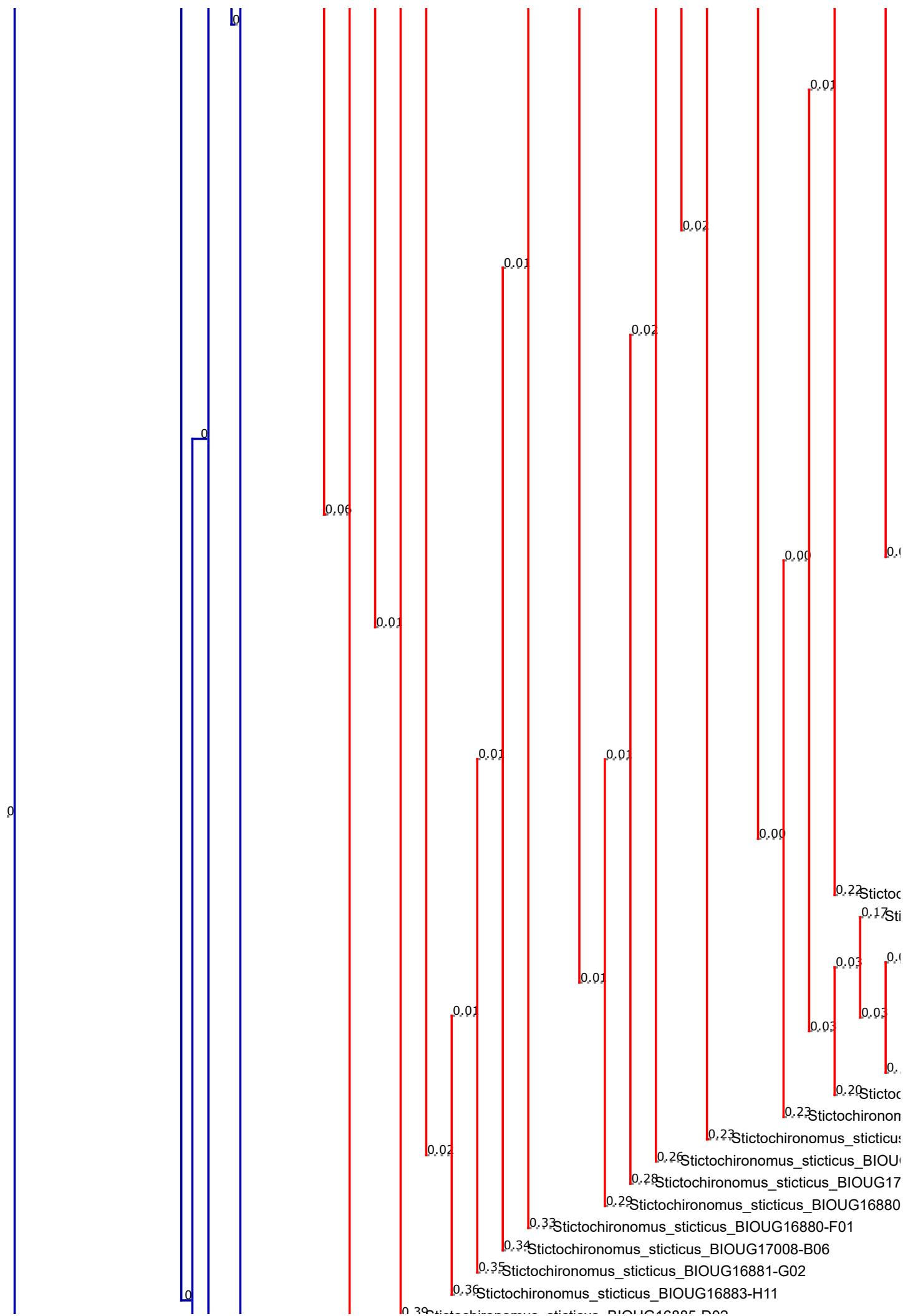

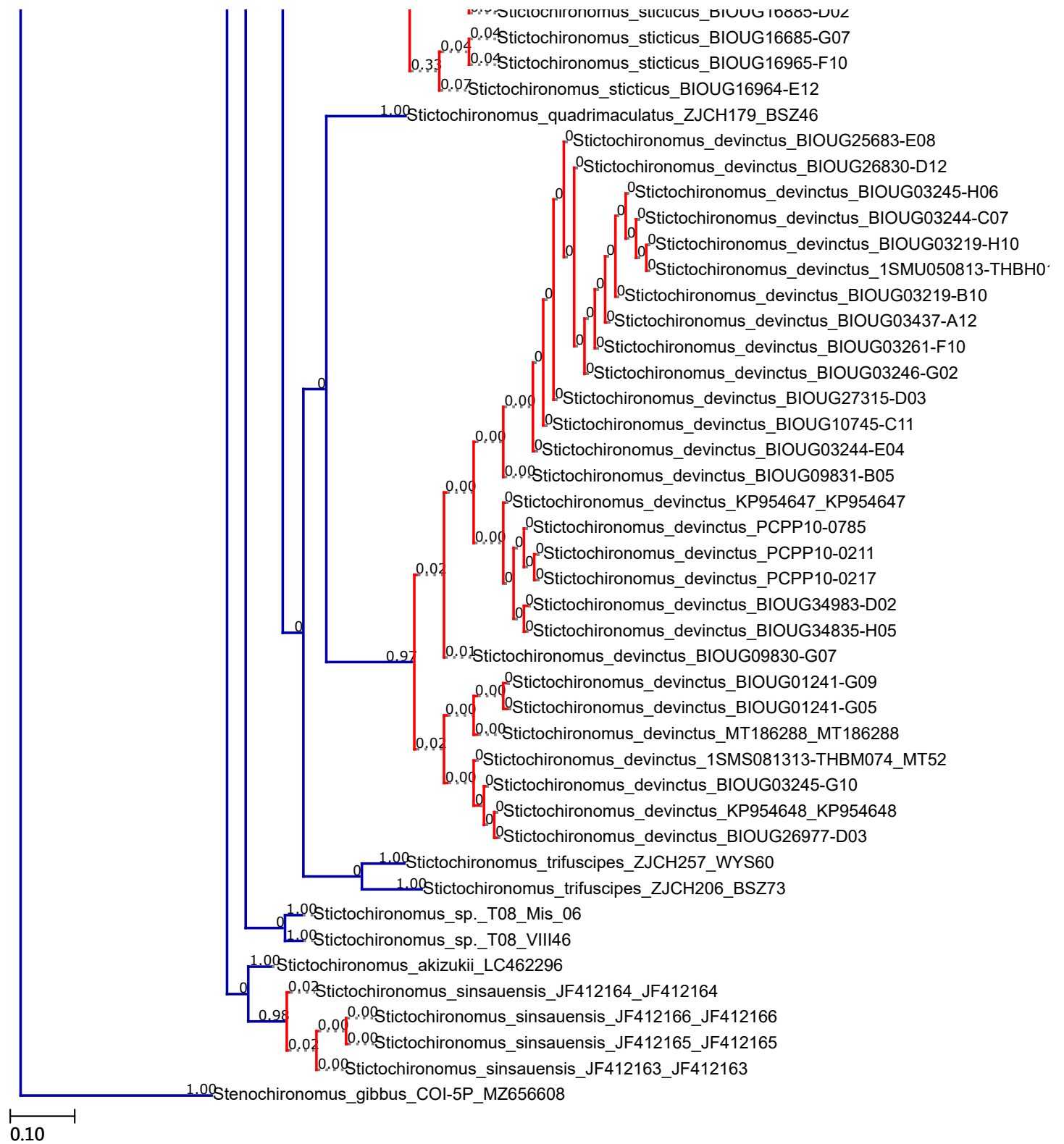

Supplement: Supplementary file 1 [file insects-15-00179-s001.zip › insects-2882236-supplementary/S11.pdf]

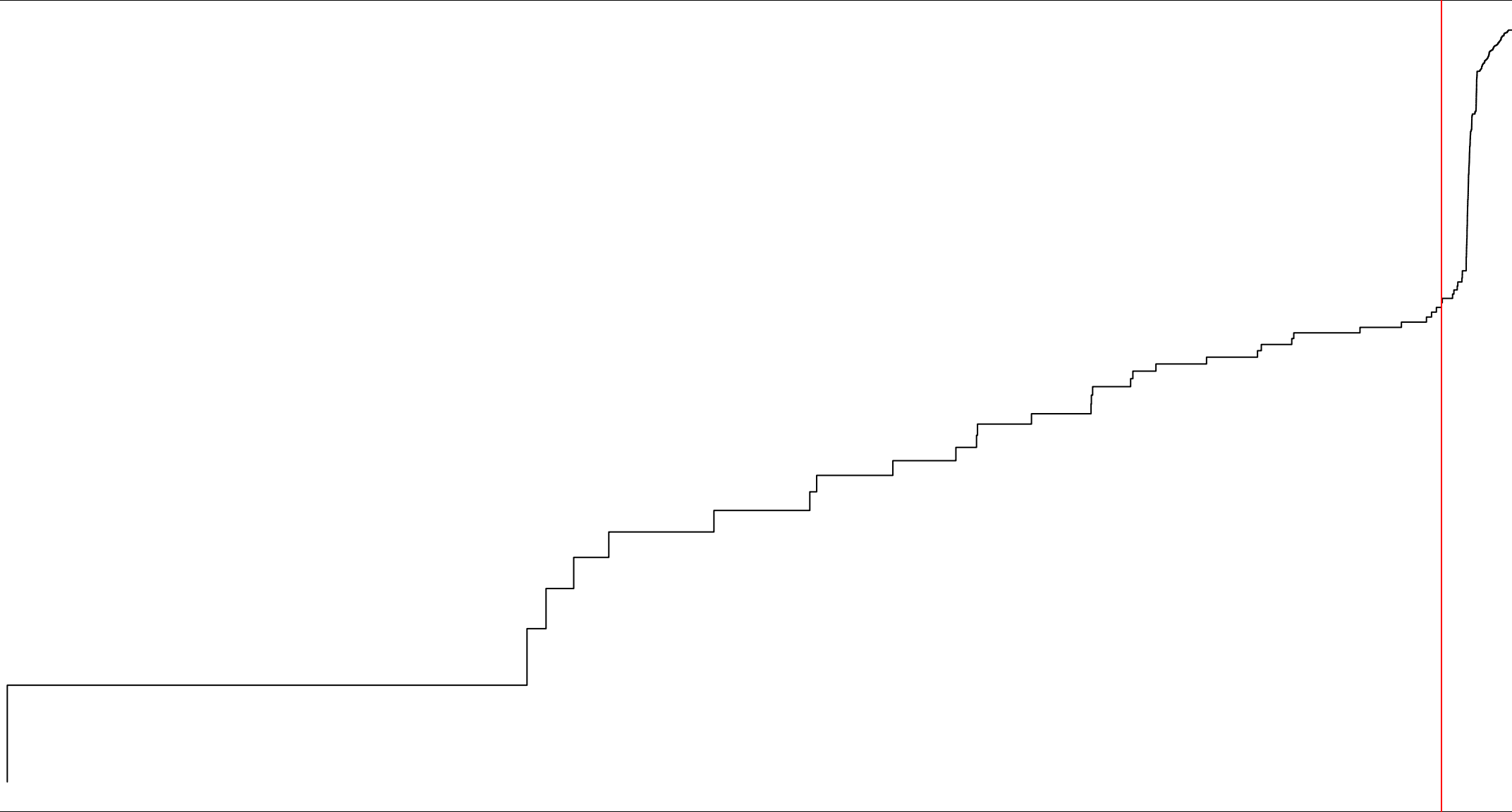

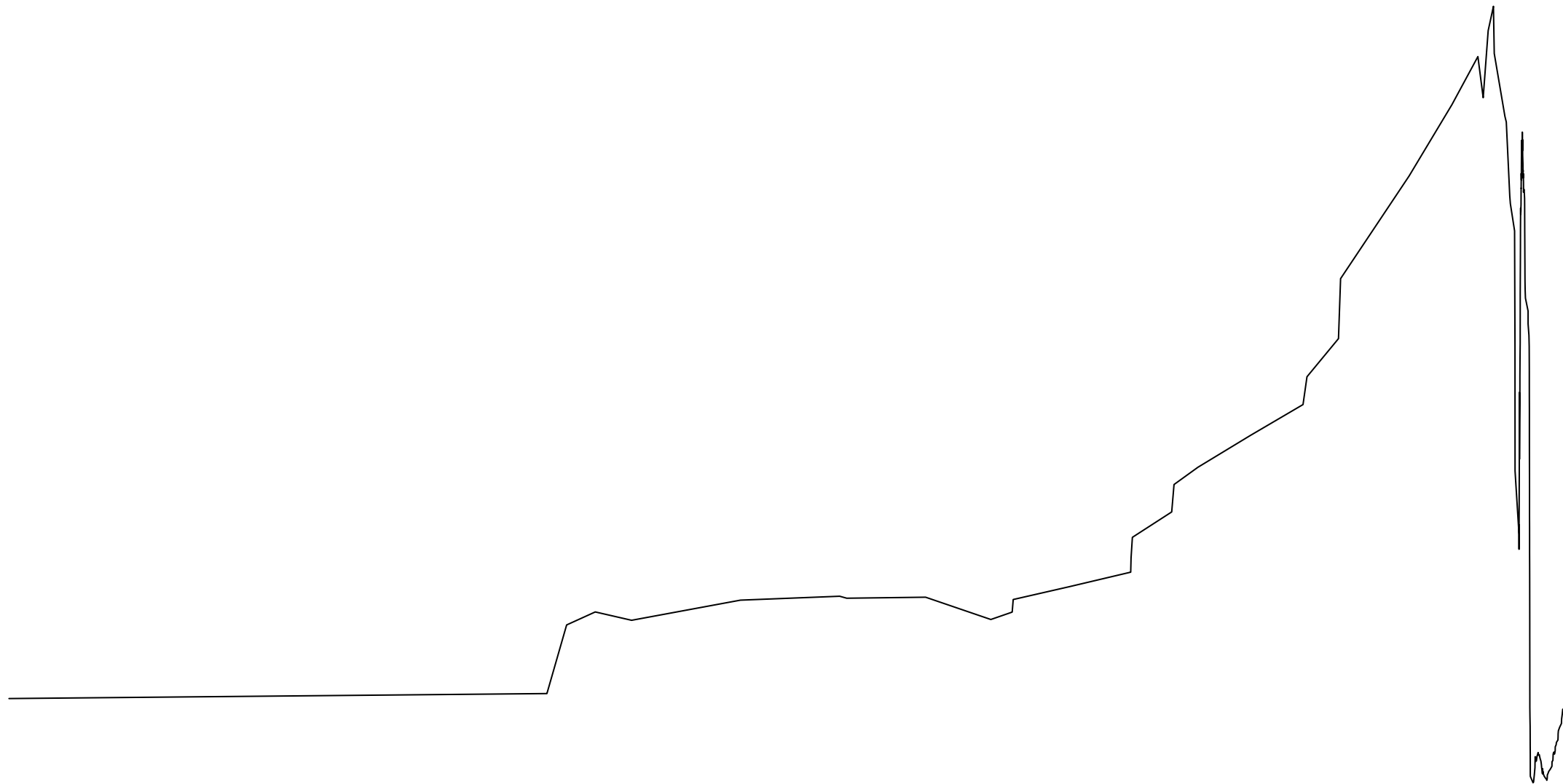

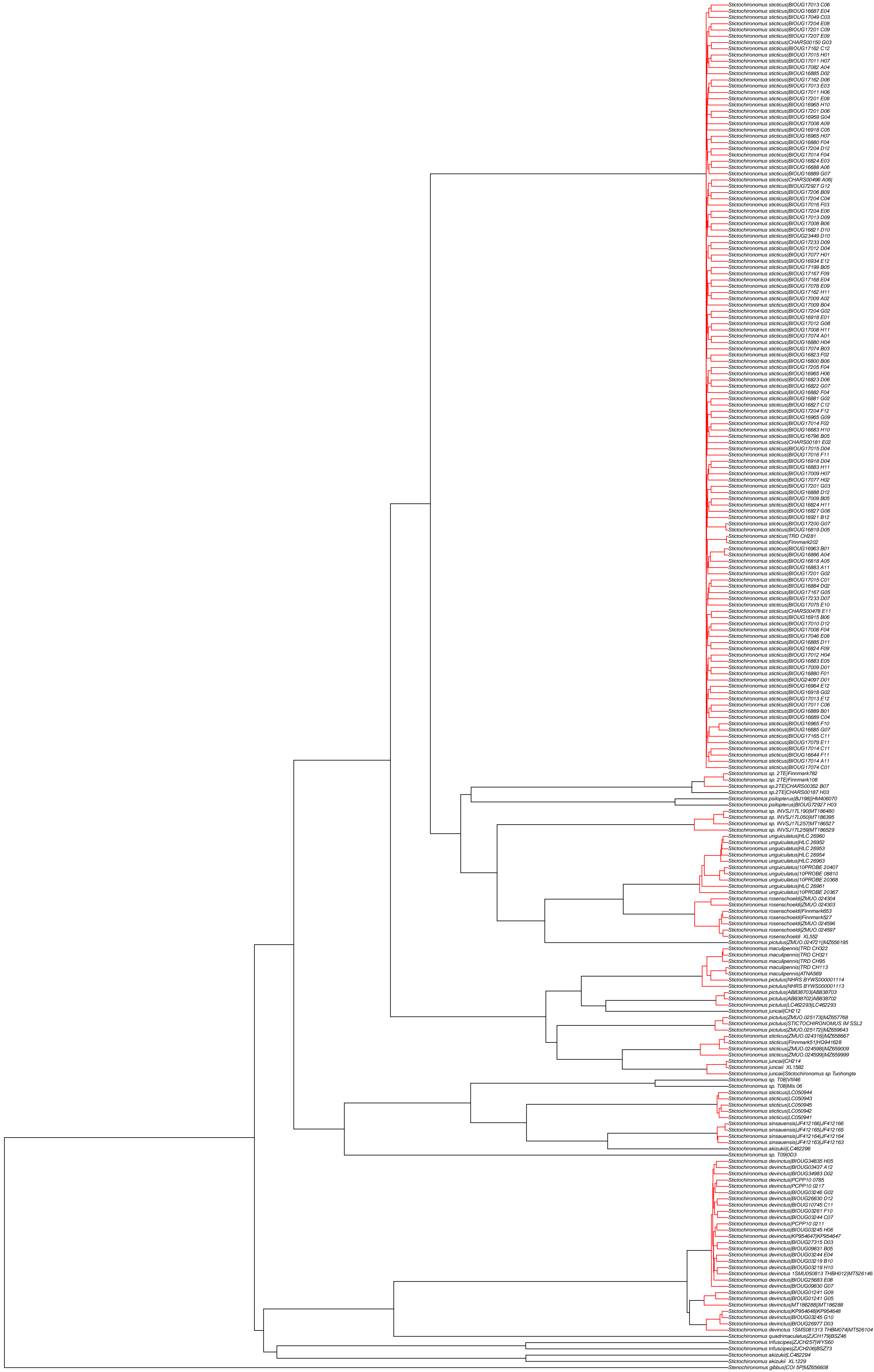

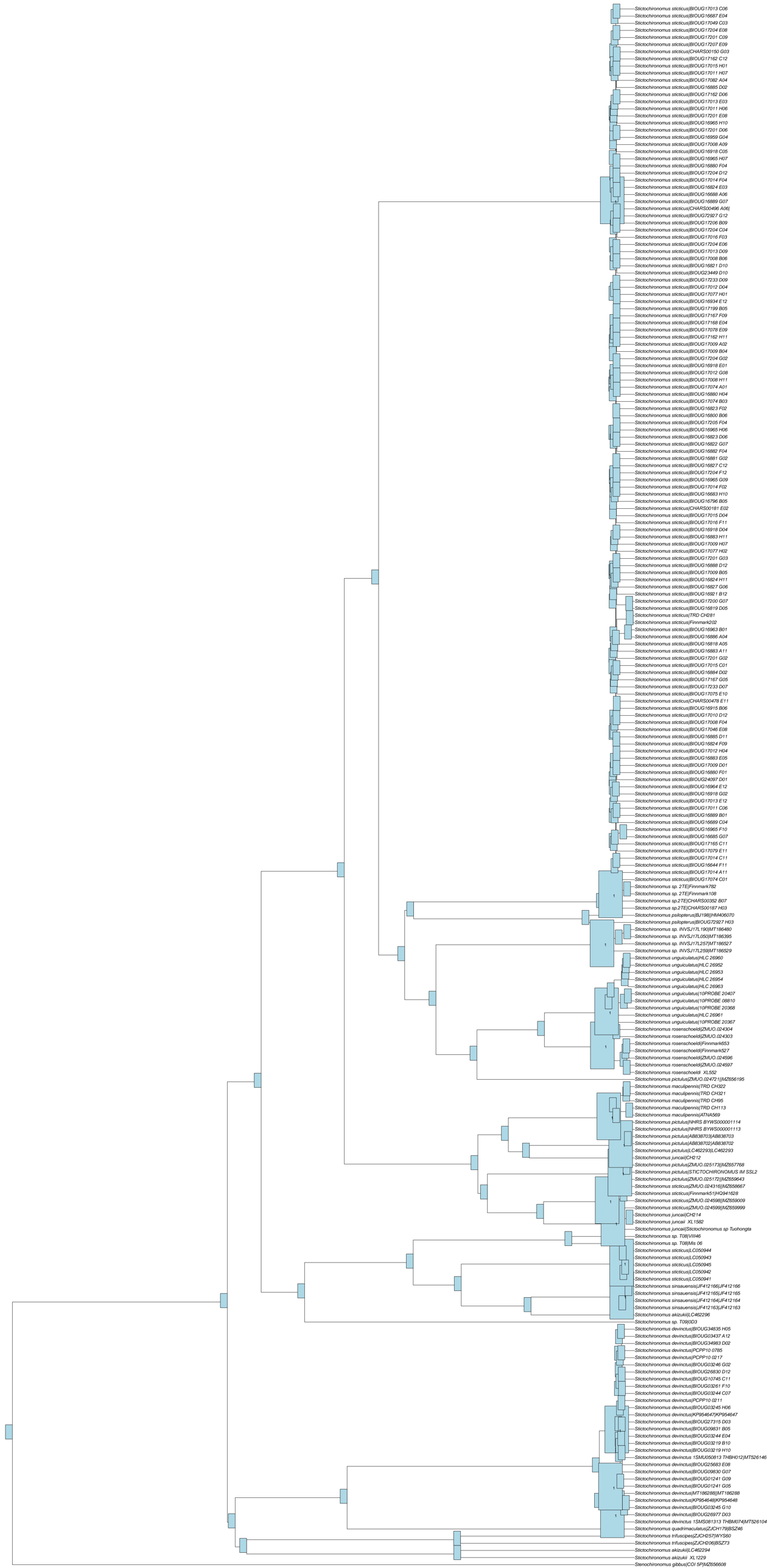

Supplement: Supplementary file 1 [file insects-15-00179-s001.zip › insects-2882236-supplementary/S12.pdf]

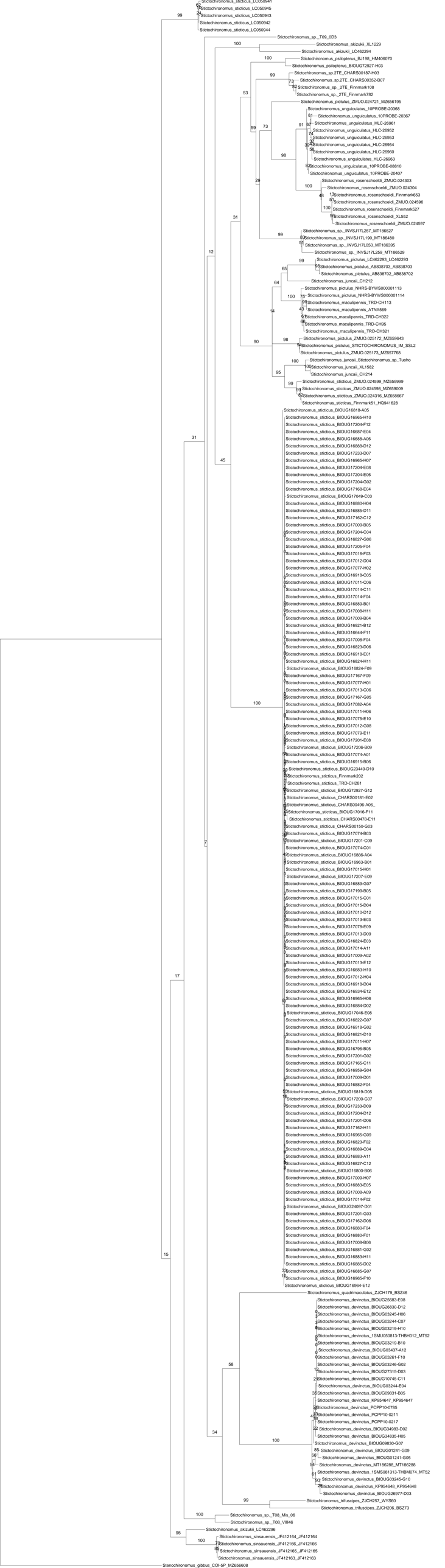

Supplement: Supplementary file 1 [file insects-15-00179-s001.zip › insects-2882236-supplementary/S7.pdf]

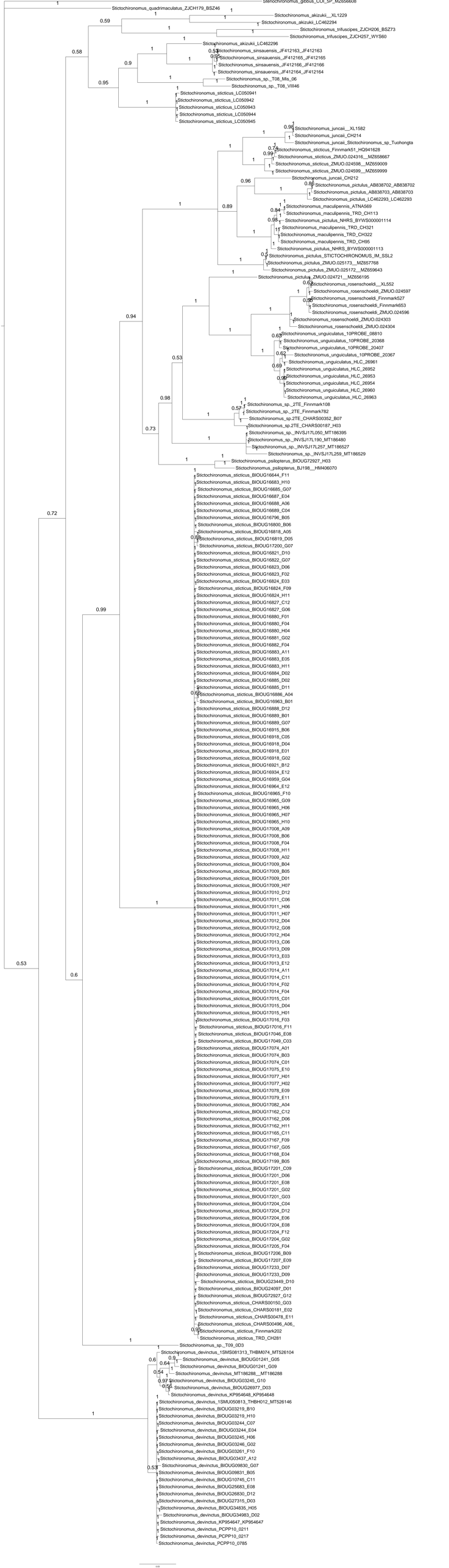

Supplement: Supplementary file 1 [file insects-15-00179-s001.zip › insects-2882236-supplementary/S8.pdf]
