# Supplementary material for: DNA Barcoding Supports “Color-Pattern’’-Based Species of Stictochironomus from China (Diptera: Chironomidae)
Source: Insects. 2024 Mar 6;15(3):179. doi: 10.3390/insects15030179 (PMC10971086; doi:10.3390/insects15030179)
Supplement: Supplementary file 1 [file S6.pdf › insects-2882236-supplementary/S6.pdf]

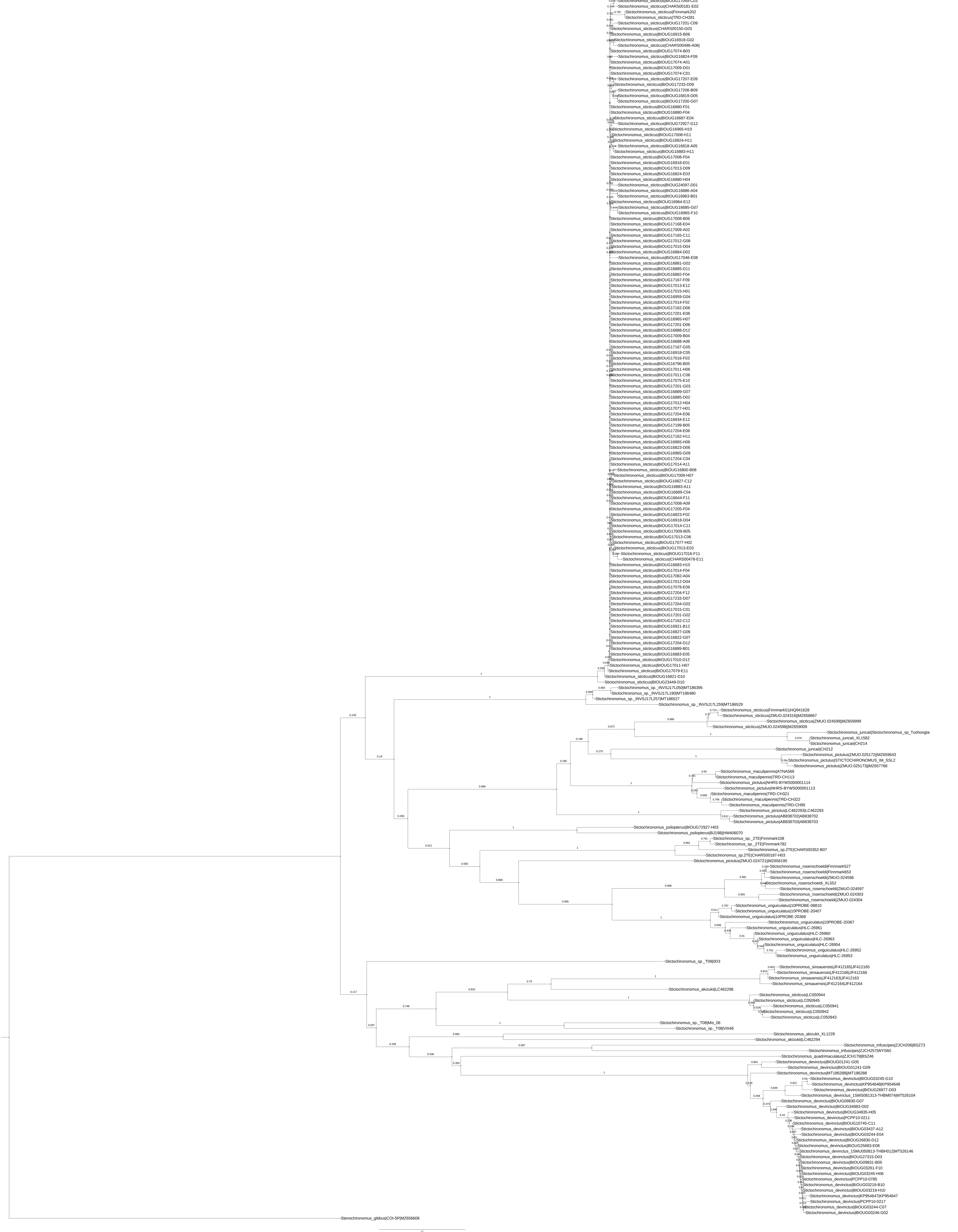

Stictochironomus_sticticus|BIOUG17049-C03
Stictochironomus_sticticus|CHARS00181-E02
Stictochironomus_sticticus|Finnmark202
Stictochironomus_sticticus|TRD-CH281
Stictochironomus_sticticus|BIOUG17201-C09
Stictochironomus_sticticus|CHARS00150-G03
Stictochironomus_sticticus|BIOUG16915-B06
Stictochironomus_sticticus|BIOUG16918-G02
Stictochironomus_sticticus|CHARS00496-A06|
Stictochironomus_sticticus|BIOUG17074-B03
Stictochironomus_sticticus|BIOUG16824-F09
Stictochironomus_sticticus|BIOUG17074-A01
Stictochironomus_sticticus|BIOUG17009-D01
Stictochironomus_sticticus|BIOUG17074-C01
Stictochironomus_sticticus|BIOUG17207-E09
Stictochironomus_sticticus|BIOUG17233-D09
Stictochironomus_sticticus|BIOUG17206-B09
Stictochironomus_sticticus|BIOUG16819-D05
Stictochironomus_sticticus|BIOUG17200-G07
Stictochironomus_sticticus|BIOUG16880-F01
Stictochironomus_sticticus|BIOUG16880-F04
Stictochironomus_sticticus|BIOUG16687-E04
Stictochironomus_sticticus|BIOUG72927-G12
Stictochironomus_sticticus|BIOUG16965-H10
Stictochironomus_sticticus|BIOUG17008-H11
Stictochironomus_sticticus|BIOUG16824-H11
Stictochironomus_sticticus|BIOUG16818-A05
Stictochironomus_sticticus|BIOUG16883-H11
Stictochironomus_sticticus|BIOUG17008-F04
Stictochironomus_sticticus|BIOUG16918-E01
Stictochironomus_sticticus|BIOUG17013-D09
Stictochironomus_sticticus|BIOUG16824-E03
Stictochironomus_sticticus|BIOUG16880-H04
Stictochironomus_sticticus|BIOUG24097-D01
Stictochironomus_sticticus|BIOUG16886-A04
Stictochironomus_sticticus|BIOUG16963-B01
Stictochironomus_sticticus|BIOUG16964-E12
Stictochironomus_sticticus|BIOUG16685-G07
Stictochironomus_sticticus|BIOUG16965-F10
Stictochironomus_sticticus|BIOUG17008-B06
Stictochironomus_sticticus|BIOUG17168-E04
Stictochironomus_sticticus|BIOUG17009-A02
Stictochironomus_sticticus|BIOUG17165-C11
Stictochironomus_sticticus|BIOUG17012-G08
Stictochironomus_sticticus|BIOUG17015-D04
Stictochironomus_sticticus|BIOUG16884-D02
Stictochironomus_sticticus|BIOUG17046-E08
Stictochironomus_sticticus|BIOUG16881-G02
Stictochironomus_sticticus|BIOUG16885-D11
Stictochironomus_sticticus|BIOUG16882-F04
Stictochironomus_sticticus|BIOUG17167-F09
Stictochironomus_sticticus|BIOUG17013-E12
Stictochironomus_sticticus|BIOUG17015-H01
Stictochironomus_sticticus|BIOUG16959-G04
Stictochironomus_sticticus|BIOUG17014-F02
Stictochironomus_sticticus|BIOUG17162-D06
Stictochironomus_sticticus|BIOUG17201-E08
Stictochironomus_sticticus|BIOUG16965-H07
Stictochironomus_sticticus|BIOUG17201-D06
Stictochironomus_sticticus|BIOUG16888-D12
Stictochironomus_sticticus|BIOUG17009-B04
Stictochironomus_sticticus|BIOUG16688-A06
Stictochironomus_sticticus|BIOUG17167-G05
Stictochironomus_sticticus|BIOUG16918-C05
Stictochironomus_sticticus|BIOUG17016-F03
Stictochironomus_sticticus|BIOUG16796-B05
Stictochironomus_sticticus|BIOUG17011-H06
Stictochironomus_sticticus|BIOUG17011-C06
Stictochironomus_sticticus|BIOUG17075-E10
Stictochironomus_sticticus|BIOUG17201-G03
Stictochironomus_sticticus|BIOUG16889-G07
Stictochironomus_sticticus|BIOUG16885-D02
Stictochironomus_sticticus|BIOUG17012-H04
Stictochironomus_sticticus|BIOUG17077-H01
Stictochironomus_sticticus|BIOUG17204-E06
Stictochironomus_sticticus|BIOUG16934-E12
Stictochironomus_sticticus|BIOUG17199-B05
Stictochironomus_sticticus|BIOUG17204-E08
Stictochironomus_sticticus|BIOUG17162-H11
Stictochironomus_sticticus|BIOUG16965-H06
Stictochironomus_sticticus|BIOUG16823-D06
Stictochironomus_sticticus|BIOUG16965-G09
Stictochironomus_sticticus|BIOUG17204-C04
Stictochironomus_sticticus|BIOUG17014-A11
Stictochironomus_sticticus|BIOUG16800-B06
Stictochironomus_sticticus|BIOUG17009-H07
Stictochironomus_sticticus|BIOUG16827-C12
Stictochironomus_sticticus|BIOUG16883-A11
Stictochironomus_sticticus|BIOUG16689-C04
Stictochironomus_sticticus|BIOUG16644-F11
Stictochironomus_sticticus|BIOUG17008-A09
Stictochironomus_sticticus|BIOUG17205-F04
Stictochironomus_sticticus|BIOUG16823-F02
Stictochironomus_sticticus|BIOUG16918-D04
Stictochironomus_sticticus|BIOUG17014-C11
Stictochironomus_sticticus|BIOUG17009-B05
Stictochironomus_sticticus|BIOUG17013-C06
Stictochironomus_sticticus|BIOUG17077-H02
Stictochironomus_sticticus|BIOUG17013-E03
Stictochironomus_sticticus|BIOUG17016-F11
Stictochironomus_sticticus|CHARS00478-E11
Stictochironomus_sticticus|BIOUG16683-H10
Stictochironomus_sticticus|BIOUG17014-F04
Stictochironomus_sticticus|BIOUG17082-A04
Stictochironomus_sticticus|BIOUG17012-D04
Stictochironomus_sticticus|BIOUG17078-E09
Stictochironomus_sticticus|BIOUG17204-F12
Stictochironomus_sticticus|BIOUG17233-D07
Stictochironomus_sticticus|BIOUG17204-G02
Stictochironomus_sticticus|BIOUG17015-C01
Stictochironomus_sticticus|BIOUG17201-G02
Stictochironomus_sticticus|BIOUG17162-C12
Stictochironomus_sticticus|BIOUG16921-B12
Stictochironomus_sticticus|BIOUG16827-G06
Stictochironomus_sticticus|BIOUG16822-G07
Stictochironomus_sticticus|BIOUG17204-D12
Stictochironomus_sticticus|BIOUG16889-B01
Stictochironomus_sticticus|BIOUG16883-E05
Stictochironomus_sticticus|BIOUG17010-D12
Stictochironomus_sticticus|BIOUG17011-H07
Stictochironomus_sticticus|BIOUG17079-E11
Stictochironomus_sticticus|BIOUG16821-D10
Stictochironomus_sticticus|BIOUG23449-D10
Stictochironomus_sp._INVSJ17L050|MT186395
Stictochironomus_sp._INVSJ17L190|MT186480
Stictochironomus_sp._INVSJ17L257|MT186527
Stictochironomus_sp._INVSJ17L259|MT186529
Stictochironomus_sticticus|Finnmark51|HQ941628
Stictochironomus_sticticus|ZMUO.024316||MZ658667
Stictochironomus_sticticus|ZMUO.024599||MZ659999
Stictochironomus_sticticus|ZMUO.024598||MZ659009
Stictochironomus_juncaii|Stictochironomus_sp_Tuohongta
Stictochironomus_juncaii_XL1582
Stictochironomus_juncaii|CH214
Stictochironomus_juncaii|CH212
Stictochironomus_pictulus|ZMUO.025172||MZ659643
Stictochironomus_pictulus|STICTOCHIRONOMUS_IM_SSL2
Stictochironomus_pictulus|ZMUO.025173||MZ657768
Stictochironomus_maculipennis|ATNA569
Stictochironomus_maculipennis|TRD-CH113
Stictochironomus_pictulus|NHRS-BYWS000001114
Stictochironomus_pictulus|NHRS-BYWS000001113
Stictochironomus_maculipennis|TRD-CH321
Stictochironomus_maculipennis|TRD-CH322
Stictochironomus_maculipennis|TRD-CH95
Stictochironomus_pictulus|LC462293|LC462293
Stictochironomus_pictulus|AB838702|AB838702
Stictochironomus_pictulus|AB838703|AB838703
Stictochironomus_psilopterus|BIOUG72927-H03
Stictochironomus_psilopterus|BJ198||HM406070
Stictochironomus_sp._2TE|Finnmark108
Stictochironomus_sp._2TE|Finnmark782
Stictochironomus_sp.2TE|CHARS00352-B07
Stictochironomus_sp.2TE|CHARS00187-H03
Stictochironomus_pictulus|ZMUO.024721||MZ656195
Stictochironomus_rosenschoeldi|Finnmark527
Stictochironomus_rosenschoeldi|Finnmark653
Stictochironomus_rosenschoeldi|ZMUO.024596
Stictochironomus_rosenschoeldi_XL552
Stictochironomus_rosenschoeldi|ZMUO.024597
Stictochironomus_rosenschoeldi|ZMUO.024303
Stictochironomus_rosenschoeldi|ZMUO.024304
Stictochironomus_unguiculatus|10PROBE-08810
Stictochironomus_unguiculatus|10PROBE-20407
Stictochironomus_unguiculatus|10PROBE-20368
Stictochironomus_unguiculatus|10PROBE-20367
Stictochironomus_unguiculatus|HLC-26961
Stictochironomus_unguiculatus|HLC-26960
Stictochironomus_unguiculatus|HLC-26963
Stictochironomus_unguiculatus|HLC-26954
Stictochironomus_unguiculatus|HLC-26952
Stictochironomus_unguiculatus|HLC-26953
Stictochironomus_sp._T09|OD3
Stictochironomus_sinsauensis|JF412165|JF412165
Stictochironomus_sinsauensis|JF412166|JF412166
Stictochironomus_sinsauensis|JF412163|JF412163
Stictochironomus_sinsauensis|JF412164|JF412164
Stictochironomus_akizukii|LC462296
Stictochironomus_sticticus|LC050944
Stictochironomus_sticticus|LC050945
Stictochironomus_sticticus|LC050941
Stictochironomus_sticticus|LC050942
Stictochironomus_sticticus|LC050943
Stictochironomus_sp._T08|Mis_06
Stictochironomus_sp._T08|VIII46
Stictochironomus_akizukii_XL1229
Stictochironomus_akizukii|LC462294
Stictochironomus_trifuscipes|ZJCH206|BSZ73
Stictochironomus_trifuscipes|ZJCH257|WYS60
Stictochironomus_quadrimaculatus|ZJCH179|BSZ46
Stictochironomus_devinctus|BIOUG01241-G05
Stictochironomus_devinctus|BIOUG01241-G09
Stictochironomus_devinctus|MT186288|MT186288
Stictochironomus_devinctus|BIOUG03245-G10
Stictochironomus_devinctus|KP954648|KP954648
Stictochironomus_devinctus|BIOUG26977-D03
Stictochironomus_devinctus_1SMS081313-THBM074|MT526104
Stictochironomus_devinctus|BIOUG09830-G07
Stictochironomus_devinctus|BIOUG34983-D02
Stictochironomus_devinctus|BIOUG34835-H05
Stictochironomus_devinctus|PCPP10-0211
Stictochironomus_devinctus|BIOUG10745-C11
Stictochironomus_devinctus|BIOUG03437-A12
Stictochironomus_devinctus|BIOUG03244-E04
Stictochironomus_devinctus|BIOUG26830-D12
Stictochironomus_devinctus|BIOUG25683-E08
Stictochironomus_devinctus_1SMU050813-THBH012|MT526146
Stictochironomus_devinctus|BIOUG27315-D03
Stictochironomus_devinctus|BIOUG09831-B05
Stictochironomus_devinctus|MT186289|MT186289
Stictochironomus_devinctus|BIOUG03245-H06
Stictochironomus_devinctus|PCPP10-0785
Stictochironomus_devinctus|BIOUG03219-B10
Stictochironomus_devinctus|BIOUG03219-H10
Stictochironomus_devinctus|KP954647|KP954647
Stictochironomus_devinctus|PCPP10-0217
Stictochironomus_devinctus|BIOUG03244-C07
Stictochironomus_devinctus|BIOUG03246-G02
Stenochironomus_gibbus|COI-5P|MZ656608
